# Evidence for GC-biased gene conversion as a driver of between-lineage differences in avian base composition

**DOI:** 10.1186/s13059-014-0549-1

**Published:** 2014-12-11

**Authors:** Claudia C Weber, Bastien Boussau, Jonathan Romiguier, Erich D Jarvis, Hans Ellegren

**Affiliations:** Department of Evolutionary Biology, Evolutionary Biology Centre, Uppsala University, Norbyvägen 18D, SE-752 36 Uppsala, Sweden; Laboratoire de Biométrie et Biologie Evolutive, Université de Lyon, Université Lyon 1, CNRS, UMR5558 Villeurbanne, France; CNRS, Université Montpellier 2, UMR 5554, ISEM, Montpellier, France; Department of Neurobiology, Howard Hughes Medical Institute, Duke University Medical Center, Durham, NC USA

## Abstract

**Background:**

While effective population size (N_e_) and life history traits such as generation time are known to impact substitution rates, their potential effects on base composition evolution are less well understood. GC content increases with decreasing body mass in mammals, consistent with recombination-associated GC biased gene conversion (gBGC) more strongly impacting these lineages. However, shifts in chromosomal architecture and recombination landscapes between species may complicate the interpretation of these results. In birds, interchromosomal rearrangements are rare and the recombination landscape is conserved, suggesting that this group is well suited to assess the impact of life history on base composition.

**Results:**

Employing data from 45 newly and 3 previously sequenced avian genomes covering a broad range of taxa, we found that lineages with large populations and short generations exhibit higher GC content. The effect extends to both coding and non-coding sites, indicating that it is not due to selection on codon usage. Consistent with recombination driving base composition, GC content and heterogeneity were positively correlated with the rate of recombination. Moreover, we observed ongoing increases in GC in the majority of lineages.

**Conclusions:**

Our results provide evidence that gBGC may drive patterns of nucleotide composition in avian genomes and are consistent with more effective gBGC in large populations and a greater number of meioses per unit time; that is, a shorter generation time. Thus, in accord with theoretical predictions, base composition evolution is substantially modulated by species life history.

**Electronic supplementary material:**

The online version of this article (doi:10.1186/s13059-014-0549-1) contains supplementary material, which is available to authorized users.

## Background

Life history traits (LHTs) and, by extension, effective population size (N_e_) have long been connected to patterns of sequence evolution. Lower body mass and shorter generation time predict rapid molecular evolution [1-5], while small-bodied animals with putatively large populations tend to show overall decreases in the d_N_/d_S_ ratio [6,7], reflecting variation in both substitution rates and selection efficiency between lineages. Meanwhile, the connection between population size, generation time, and base composition is less well understood. In principle, any life history-related trait affecting substitution patterns should also impact on the evolution and the dynamics of base composition. GC-biased gene conversion (gBGC) associated with meiotic recombination leads to the preferential fixation of GC in AT/GC heterozygotes and is a major determinant of base composition. Direct experimental evidence is currently limited to *S. cerevisiae*, with a significant 1.3% excess of transmitted GC alleles thought to result from a bias in the mismatch repair machinery [8-11]. However, evidence for its effects is observed across a wide range of taxa [12-16], leading to a widespread association between GC content and crossover rates [8,13,17-20].

Importantly, it has been suggested that LHTs predict how strong the effects of gBGC on compositional evolution are [21]. Much as recombination locally modulates N_e_ [22], N_e_ is in turn predicted to increase the efficacy of gBGC in the same manner that it increases the efficacy of selection. This is because GC alleles behave as though they were positively selected when their fixation is favored and the effect of drift will decrease with increasing N_e_ [15,23]. Species with short generation times additionally experience a greater number of meioses per unit time, and therefore more frequent gBGC. In agreement with this idea, Romiguier *et al.* [21] observed that mammalian lineages show negative correlations between both body mass, expected to be negatively associated with N_e_, and generation time and GC. Subsequent studies on mammals have reinforced these findings [24,25].

The interpretation of these results may however be complicated by differences in chromosomal architecture between species under comparison. Large-scale chromosomal rearrangements may obscure the impact of LHTs on base composition by shifting the recombination landscape [26,27]. Rodents, which exhibit weaker gBGC than primates despite having larger populations, are a striking example. This is thought to be a consequence of having lengthened chromosome arms and reduced crossover rates due to a shift to acrocentric centromeres [28]. Additional studies in different clades are therefore necessary to disentangle the effects of changes in the recombination map and changes in population size on GC content evolution [25].

Here, we explore the idea that some of the caveats associated with changes in chromosomal architecture might be avoided by studying birds. The avian karyotype comprises a large number of chromosomes (haploid count = 39 for chicken, typical for most birds) with a remarkably low rate of interchromosomal rearrangement between species [29-32]. For instance, despite a split time of 84 to 94 million years (My, reported in our companion phylogenomic study [33]), the karyotypes of chicken and zebra finch differ merely by one fission and one fusion event [34]. Accordingly, we expect the stability of the avian karyotype to translate to greater stability in broad-scale recombination landscapes over time. This is empirically supported by a correlation in the rate of recombination in 1 Mb windows between homologous regions of chicken and zebra finch chromosomes [19]. Additionally, birds lack a functional copy of *PRDM9* [35], which is expected to reduce shifts in the recombination landscape associated with rapid hotspot turnover [36,37]. As a consequence, between-lineage variation in composition should be owing to differences in LHTs rather than genome architecture. Meanwhile the effects of recombination on a given sequence will have remained consistent throughout its history, and are therefore expected to leave clear signatures [36,38].

Avian genomes show considerable variation in chromosome size, with the majority of chromosomes being small micro-chromosomes. Given the requirement for at least one crossover per chromosome [39], this results in high crossover rates [40-42]. Signatures of gBGC ought to be most readily detected in lineages with more fragmented karyotypes, that is, many small chromosomes [16,24]. Indeed, the continuing reinforcement of intragenomic heterogeneity in GC content appears to be particularly pronounced in chicken [43], unlike in some mammals where erosion of GC-rich regions has occurred [15,21,28,44-46]; these studies have excluded CpG sites in the analyses so there is a remaining issue how such sites influence the evolution of base composition. Finally, base composition varies greatly between different bird lineages [47].

Thus, birds have several features that make them especially interesting for investigating the interactions between recombination, selection, base composition and substitution rates. A recent effort that sequenced 45 whole bird genomes along with three previously published ones (48 total, see Additional file 1) covering all major avian orders now provides the opportunity to investigate these questions [48]. The availability of orthologous coding and intronic sequences from these species allows us to examine trends that may be less apparent with fewer sequences or taxa. Here, we focus on the impact of between-lineage differences in effective population size and time-scaled recombination rates on base composition in birds, and test whether gBGC might explain the substantial variation in GC content observed. We first ask whether there is a significant negative association between LHTs and GC content, and find this to be the case. We also test how robust this result is by employing alternative proxies of N_e_ based on phylogenetic discord among gene trees. We then consider the degree to which different classes of sites are affected, how it corresponds to recombination rate estimates, and whether the impact of gBGC on the base composition of avian genomes is ongoing.

## Results

### Correlation between GC3 and life history traits is consistent with stronger gBGC in large populations with short generation times

**Figure 1:**
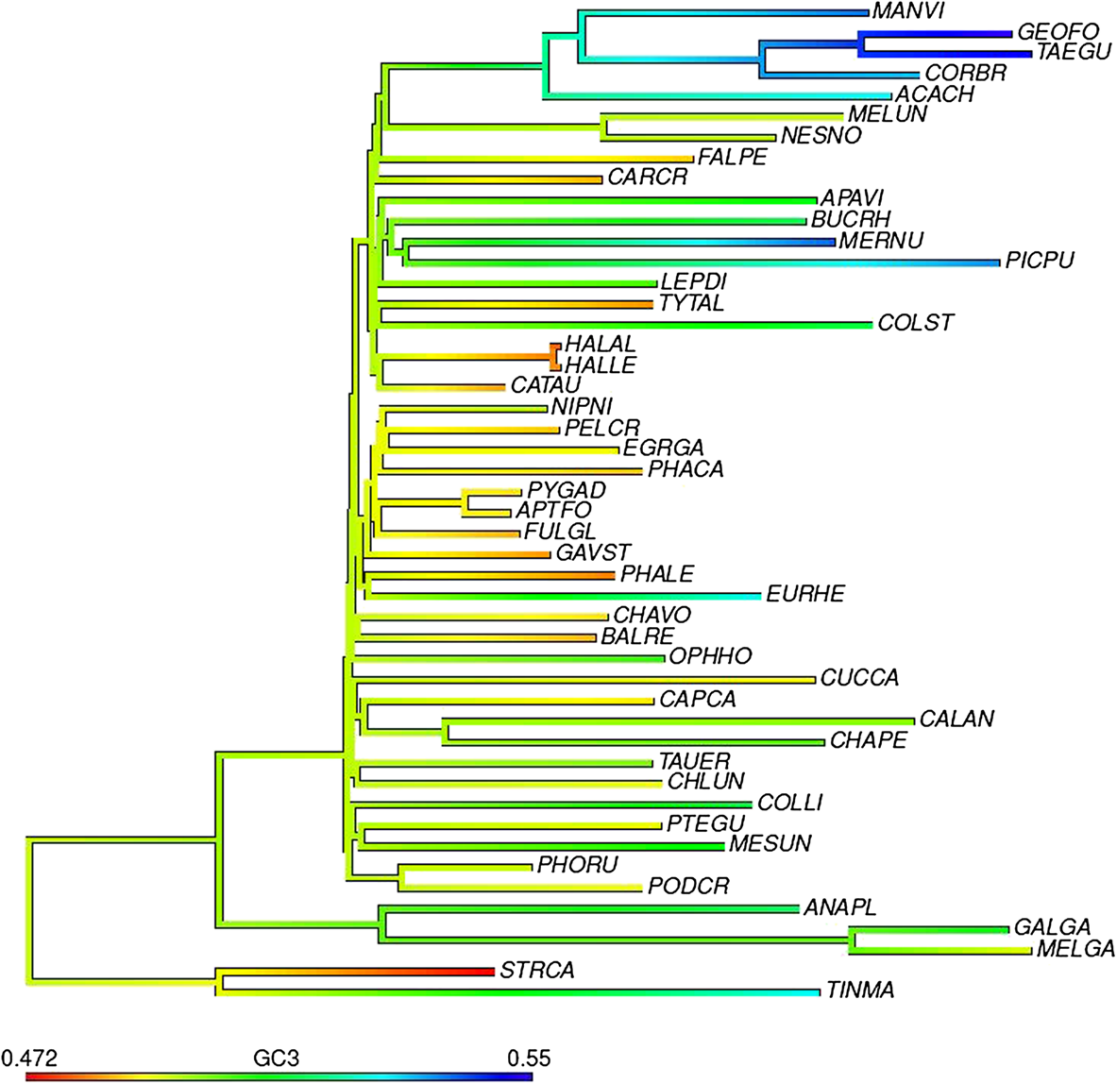
**GC3 content varies substantially between different avian lineages.** Total evidence nucleotide tree [33] showing differences in GC3 content between lineages. Ancestral GC3 was estimated by ML using contMap from R phytools for illustration. See Additional file 1 for species names.

**Figure 2:**
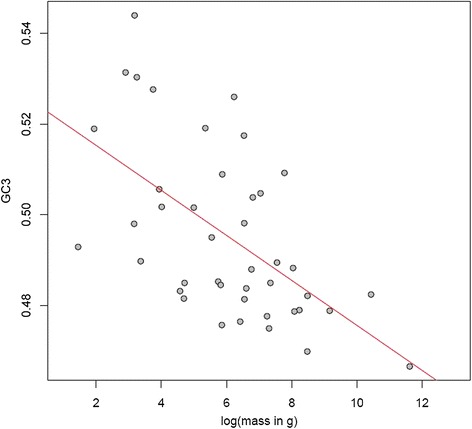
**GC3 content is significantly negatively correlated with body mass.** Small-bodied birds have elevated GC3 content compared to larger-bodied species.

Given the substantial heterogeneity in GC3 content (the proportion of GC at third codon positions) between avian species [33,47] (Figure 1), we asked whether there is evidence that third codon sites, which should be the least constrained coding positions, might be subject to the influence of recombination-associated gBGC. Species with smaller body mass are expected to have both shorter generation times and larger effective population sizes, increasing both the number of meioses per unit time and the efficacy of gBGC [21,23]. If gBGC is a factor in determining GC, small-bodied species ought then to have elevated GC. This is indeed what we observed, with species with greater body mass exhibiting lower GC3 than species with smaller body mass (Spearman’s rho = -0.5866, *P* = 6.2e-05, *n* = 42; see Figure 2). Despite the limited number of species for which data are available, maximum longevity (rho = -0.3645, *P* = 0.0616, *n* = 27) and age of first female sexual maturity (rho = -0.5957, *P* = 0.0071, *n* = 19) showed similar trends, consistent with the possibility that short generation times lead to an increase in GC3 assuming equilibrium has not yet been reached. In the following we only examine body mass, as this maximizes the number of species we can consider.

### Nucleotide composition at both coding and non-coding sites is predicted by body mass

Selective constraint and mutational and neutral forces acting on base composition interact with each other and modulate to what extent the composition at a given class of site varies. In species with large effective population sizes, selection against weakly deleterious mutations is more efficient, which can lead to synonymous sites being constrained, for instance due to selection on translational efficacy [49]. According to theory, this trend would be further exacerbated by locally increased N_e_ in regions of high recombination, owing to the increased efficacy of selection [22]. However, support for increased levels of codon usage bias in highly expressed genes is thus far absent in birds [50,51] and weak if at all present in mammals [51-55].

To nevertheless rule out the possibility that our findings can be accounted for by selection on synonymous sites, we assessed whether intronic sequences are similarly negatively correlated with body mass and found this to be the case (rho = -0.4411, *P* = 0.0038). Selection on mRNA folding tends to be enhanced by high GC content and is thus suggested to constrain sequence evolution [56]. However, as this is hypothesized to relate to translational efficiency [56,57] only mature mRNA structure is relevant. Thus, intronic GC content ought not to be subject to constraint in this respect, and gBGC is a plausible explanation for the pattern observed.

**Figure 3:**
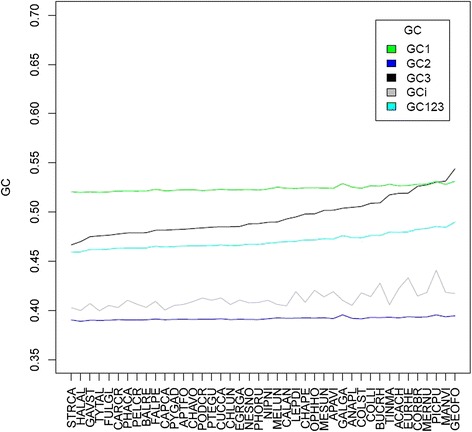
**GC3 is more heterogeneous between species than GC1 and GC2.** GC content for first, second, and third codon positions and introns (GCi). Species were ordered from left to right by ascending GC3 content. See Additional file 1 for species names.

As it is well-established that gBGC influences both synonymous and non-synonymous coding positions [58], we next tested whether the relationship between body mass and GC3 can be generalized to first and second codon positions. As expected, GC1 (rho = -0.5631, *P* = 0.0001) and GC2 (rho = -0.5639, *P* = 0.0001) show significant negative correlations with body mass that are of a similar magnitude to that observed for GC3. However, as one would predict if first and second coding positions are under stronger selective constraint than third positions, the range of GC values is substantially narrower (sd = 0.003 for GC1, sd = 0.0015 for GC2, sd = 0.0189 for GC3; see Figure 3). Owing to the structure of the genetic code, differences in GC2 between species ought to be associated with slight changes in amino acid usage between lineages. This is indeed observed (Additional file 2) and in agreement with previous observations that amino acid usage correlates with base composition [59].

The fact that first, second, and third coding sites as well as intronic sites exhibit correlations with body mass is consistent with a pervasive influence of gBGC on base composition, while the strength of the effect on a given class of site appears to be modulated by the degree of selective constraint. These relationships are not explained by phylogenetic inertia, as controlling associations between body mass and GC at different classes of sites for phylogeny did not render the correlations non-significant (Additional file 3).

### Orthologs with high between-species GC heterogeneity show a stronger effect of gBGC on base composition

In addition to treating different classes of sites separately, we can distinguish between orthologs that show high or low levels of compositional heterogeneity (that is, variance in GC) among species when addressing the relationship between gBGC and LHTs. Such heterogeneity is expected to be most pronounced in sequences that are differentially affected by recombination-associated GC fixation bias due to differences in generation time and N_e_; that is, sequences in highly recombining regions. Conversely, sequences experiencing little recombination overall should be more homogenous between species. When only genes whose third sites do not strongly reject the homogenous TN93 + GAMMA model in favor of the non-stationary model of Galtier and Gouy [60], hereafter referred to as ‘homogenous’ genes, were considered (*n* = 310) the correlation between body mass and GC3 became modestly weaker (rho = -0.4563, *P* = 0.0026) than when considering ‘non-homogenous’ (*n* = 1,470) genes (rho = -0.5887, *P* = 5.7e-05).

**Figure 4:**
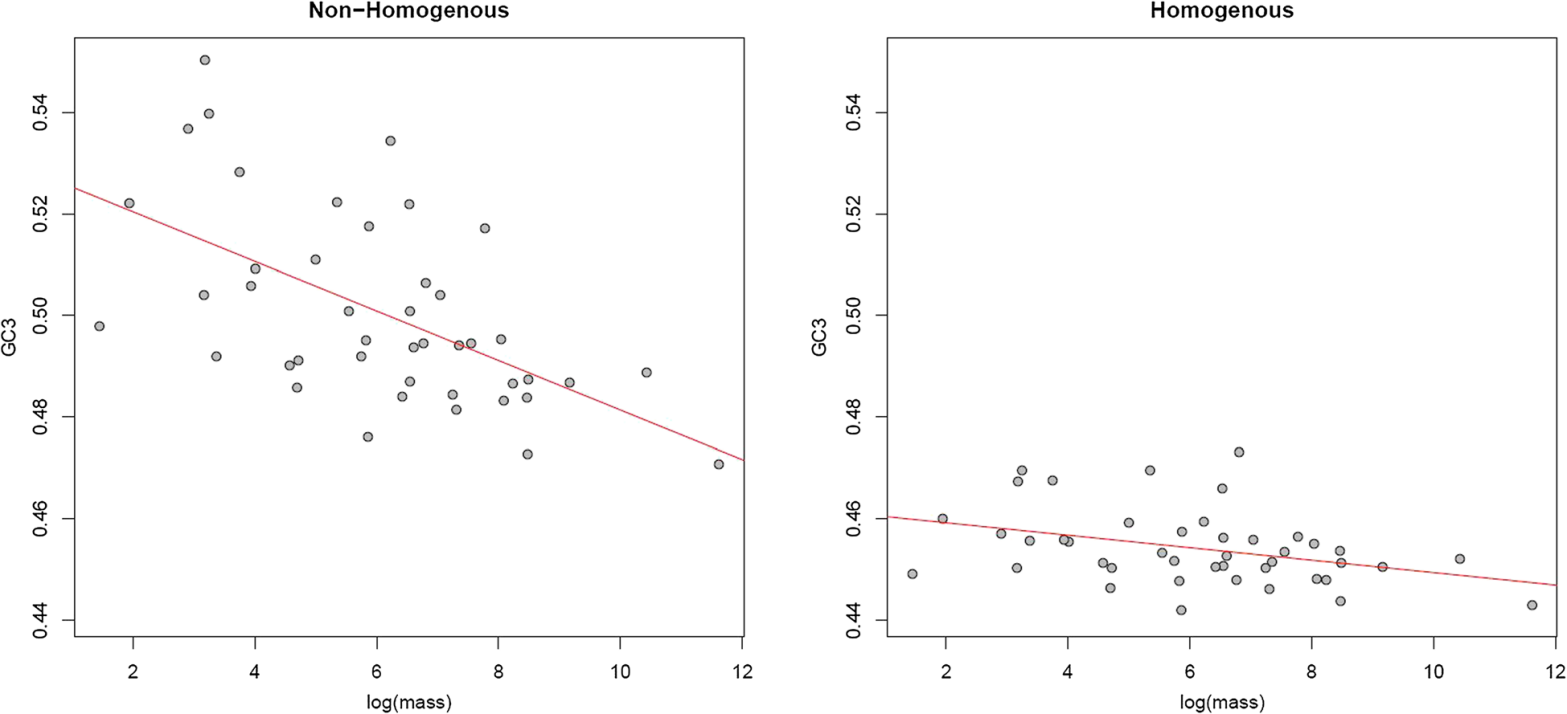
**GC3 is more strongly associated with body mass for genes with non-homogenous between-species composition.**

The difference in the strength of the correlation is not owing to sample size, as none of 10,000 randomizations where 310 non-homogenous genes were randomly sampled gave a correlation between GC3 and body mass that was equal to or weaker (that is, less negative) than that observed for the homogenous set. However, given that both the standard deviation and mean of GC3 are lower for homogenous genes (sd = 0.0084, mean = 0.4565) than for non-homogenous genes (sd = 0.0201, mean = 0.4991), the smaller correlation coefficient is expected, particularly if a low and less heterogeneous GC3 is indicative of gBGC having a weaker influence on these sequences. That there is nevertheless a relationship between GC3 and body mass for homogenous genes, albeit with a shallower slope (see Figure 4), cautions against the assumption that gBGC has no influence at all where the homogenous model is not rejected.

**Figure 5:**
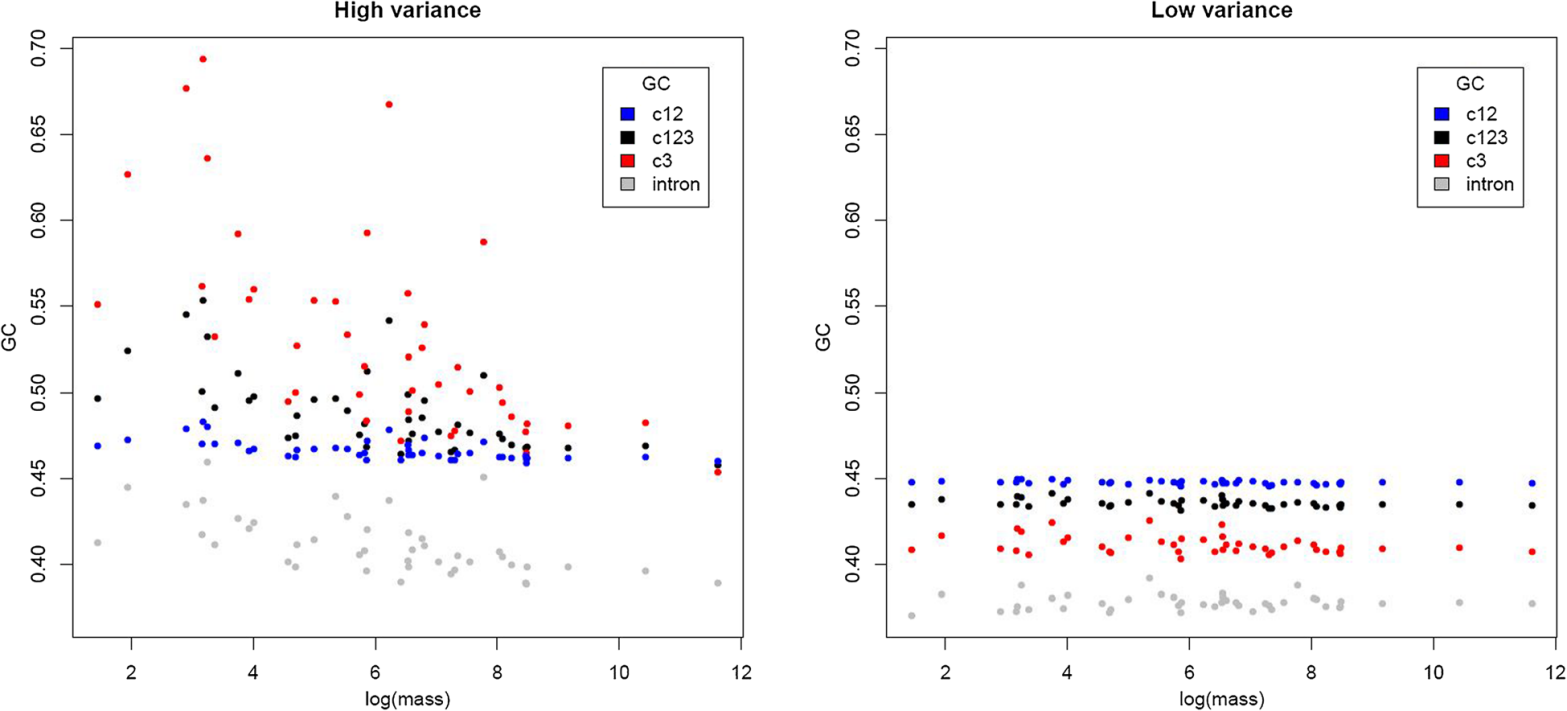
**Negative associations between coding GC and body mass are stronger for high GC3-variance orthologs.** Both high- and low-variance orthologs exhibit significant negative correlations between GC at multiple classes of coding site and body mass, although the pattern is markedly weaker in the low-variance set.

Additionally, we compared correlations between the 830 orthologs with the highest and lowest variance in GC3 [46], respectively, and obtained similar results. GC12 (rho = -0.6604, *P* = 2.8e-06), GC123 (rho = -0.6965, *P* = 6.3e-07), and GC3 (rho = -0.7057, *P* = 4.4e-07) were significantly negatively correlated with body mass for high-variance orthologs. The somewhat stronger correlation for GC3 is expected given the above definition of ‘high variance’. Low-variance orthologs showed a similar but weaker pattern. GC3 had the weakest correlation for the low-variance set (rho = -0.3138, *P* = 0.0409), as expected given its reduced heterogeneity between species (Figure 5). GC12 (rho = -0.3935, *P* = 0.0095) and GC123 (rho = -0.357, *P* = 0.0193) were somewhat more strongly correlated, but less so than for high-variance genes (Figure 5).

Introns associated with the high variance orthologs showed significant correlations between GC content and body mass (rho = -0.6451, *P* = 5.3e-06; Figure 5), whereas those associated with low-variance orthologs showed no significant correlation (*P* = 0.4378). These trends are consistent with base composition of introns of high- and low-variance genes evolving in a manner similar to the associated coding sequences, but with a weaker impact on the non-coding sequences.

### High between-species variance is driven by GC evolution in small-bodied birds

**Figure 6:**
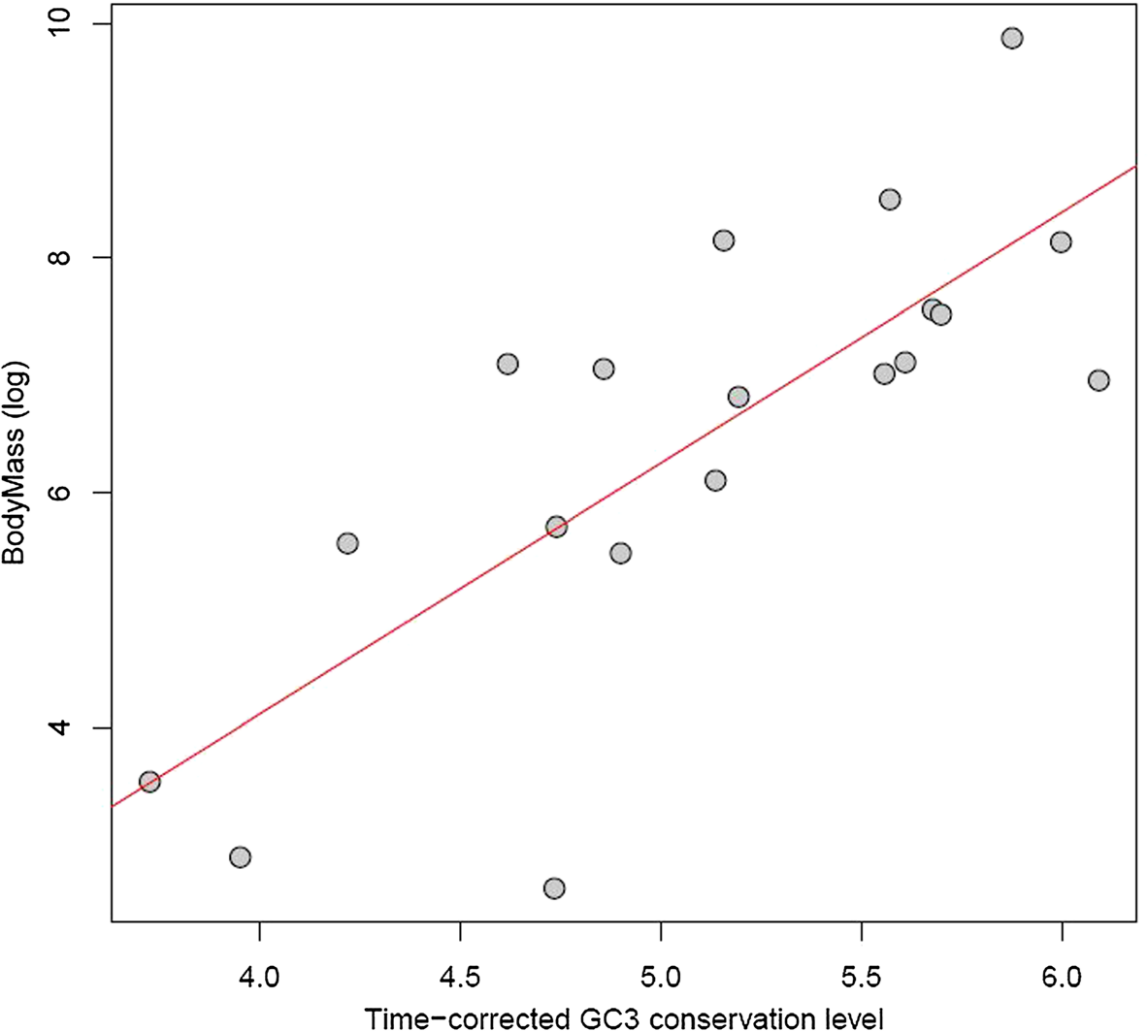
**GC3 is more conserved between pairs of large-bodied species.** Each point on the plot represents one species pair. GC3 is less conserved between genes evolving in small-bodied species pairs (see Additional file 6). The hummingbird/swift (CALAN/CHAPE) pair appears to be an outlier with high conservation (approximately 4.75), possibly owing to hummingbird having a low, and therefore more conserved, GC for its size (GC: 0.4929, body mass 4.25 g).

In order to test if high GC3 variance orthologs were produced by increases of GC3 in small-bodied species or decreases of GC3 in large-bodied species, we computed a time-corrected index of GC3 conservation for 19 pairs of species (following [61], see Materials and Methods). We again retrieved strong correlations with body mass when we considered this measure of GC3 dynamics instead of average GC3. GC3 conservation was higher between pairs of large-bodied species than pairs of small-bodied species (rho = 0.72, *P* = 8.2e-04; Figure 6) in agreement with predictions and with previous results in mammals [61]. GC-content heterogeneity between species is presumably due to increased gBGC in small-bodied species, whereas the GC-content of a gene tends to remain similar when evolving in large-bodied lineages.

### GC correlates with multiple proxies of population size

While body mass is a convenient measure to consider in absence of data on actual effective population sizes, correspondence between the two may not necessarily be strong in birds [62]. To ensure that our findings are robust, we considered an additional approach. Shared ancestral polymorphisms can lead to disagreement between gene and species trees for closely related lineages, particularly when N_e_ is large. We may therefore estimate population size by using information on discordance between gene and species trees to compute coalescent-based internal branch lengths representing ‘the quotient of the number of generations that elapsed between the more ancient divergence and the more recent divergence, and the haploid population size N’ [63] (see Materials and Methods).

**Figure 7:**
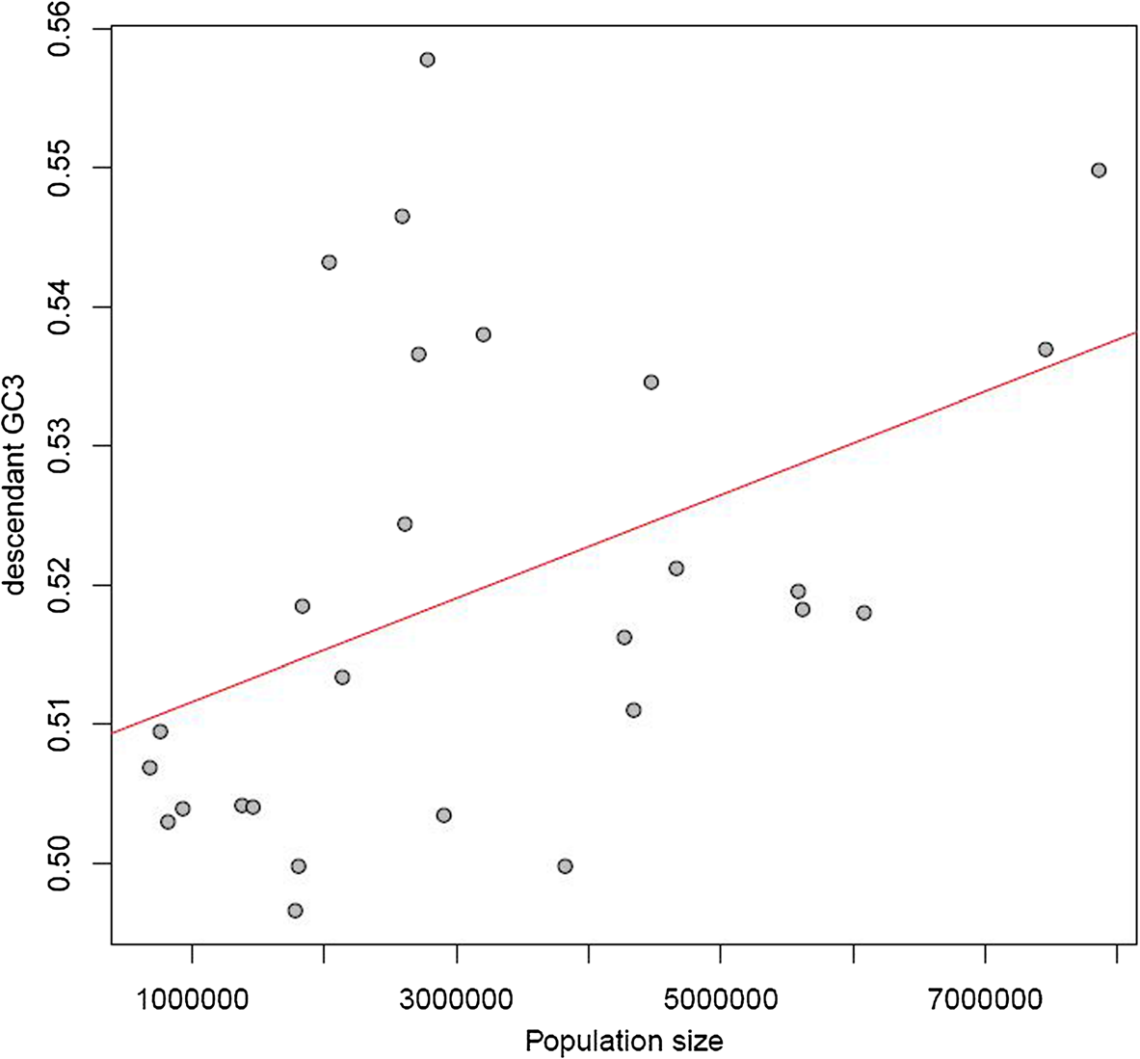
**Ancestral population size predicts descendant GC3.** Reconstructed population sizes for internal branches with two descendant lineages are positively correlated with descendant GC3 content. Population size was inferred from relationships between ancestral generation time, split dates, and coalescent branch lengths computed from the degree of gene tree discordance. Note that the population sizes here are expected to be overestimates, as age of first female sexual maturity was used as a proxy for generation time (see Materials and Methods), and are intended to be interpreted in terms of rank order.

We examined correlations between the average GC3 for the descendants of a given branch and inferred population size for said branch. Only branches giving rise to two terminal branches were considered, as effective population sizes cannot be determined for terminal branches, and are unreliable for deeper branches, which tend to be very short in this data set. If large ancestral population size is predictive of higher GC in the descendants, we expect a positive correlation between N and GC3. This is indeed what we observe regardless of the method of branch length estimation (rho = 0.3041, *P* = 0.0856 for our method; rho = 0.3471, *P* = 0.0522 for MP-EST). These correlations contain several outliers with extremely large inferred population sizes, which may be explained by errors in the topologies of the gene trees, due to low signal in gene sequences. Accordingly, when we restrict our analysis to branches with a length of ≥0.1 coalescent units the positive correlation becomes more robust (rho = 0.4963, *P* = 0.0092 for our method; rho = 0.5233, *P* = 0.0048 for MP-EST; Figure 7). Similar results were obtained when only branches where bootstrap support values for the associated nodes are ≥50 were considered (rho = 0.5583, *P* = 0.0064). Given the long time period that has elapsed since the putative ancestral populations were alive, descendant GC3 may not adequately reflect their base composition. We therefore also examined the correlation between the mean of the reconstructed equilibrium GC (GC3*) values at both ends of a given branch and N, which yielded a similar positive correlation (rho = 0.4726, *P* = 0.0277 for branches with coalescent length ≥0.1 and bootstrap ≥50).

The Coevol approach [64], which we used to estimate ancestral generation time for the above calculations and which makes use of associations between substitution patterns and life history traits (see Materials and Methods), also allows correlations between trait data and base composition through time to be examined. Results for 10 concatenated alignment blocks were qualitatively consistent with the above, namely a negative correlation between age of first female sexual maturity (used to approximate generation time) and GC, although not all reconstructions reached significant posterior probabilities (Additional file 4).

### Recombination rates in chicken and zebra finch correlate with heterogeneity in GC

The above results indicate that base composition is robustly associated with life history traits, and presumably population size, in agreement with the idea that the impact of gBGC is most pronounced in species with high N_e_ and short generation times. However, to establish a crossover-related process as a driver of between-species variation in GC content, we need to assess how compositional differences between lineages relate to meiotic recombination. If gBGC is a major driver of base composition, GC should be overall higher in regions of high recombination, which has indeed been established in multiple species [8,13,17-20]. In these regions, we might also expect GC to increase more rapidly in species where recombination occurs more frequently per unit time than in those with long generation times, resulting in greater heterogeneity between orthologs of the same sequence. It has recently been suggested that the avian ancestor was large compared to extant species [7], perhaps indicative of a lower baseline GC content. However, even if the ancestral genome was not GC-poor, high N_e_ ought, in theory, to lead to more efficient gBGC in small-bodied species counteracting the effects of the AT-biased mutation process on sequence composition [65]. In contrast, GC would decline in species with reduced N_e_. Under both scenarios, composition will be heterogeneous between species in highly recombining regions, while composition in regions of low recombination should be dominated by other forces such as mutation bias.

The absence of recombination maps for the majority of the 48 bird species limits our ability to test whether sequences located in regions of relatively high recombination show more pronounced between-species heterogeneity in GC content. We can, however, consider recombination maps from chicken [20] and zebra finch [19], two distantly related lineages, across 1 Mb windows and test for a relationship between standard deviation in GC3 and crossover rate. In accord with recombination driving increased heterogeneity in GC3, we find that both chicken and zebra finch recombination rates show a positive relationship with the between-species standard deviation in GC3 for each of 1,780 orthologs found across all species (Table 1). This cannot simply be owing to the mean and variance being coupled, which we have no *a priori* reason to expect, as regions where AT is high will have reduced rather than increased heterogeneity in AT between species.

**Table 1:** **Correlations between standard deviation in GC and median GC and recombination**

**Correlation with recombination**	**Standard deviation of GC**		**Mean GC**	
Chicken				
GC3	rho = 0.2432	*P* = 2.3e-09	rho = 0.3283	*P* = 3.0e-16
GC3 ‘homogenous’	rho = 0.2136	*P* = 0.0016	rho = 0.2665	*P* = 7.6e-05
GC i	rho = 0.2371	*P* = 2.0e-05	rho = 0.3761	*P* = 4.4e-12
Zebra finch				
GC3	rho = 0.2915	*P* = 4.7e-11	rho = 0.2689	*P* = 1.4e-09
GC3 ‘homogenous’	rho = 0.2009	*P* = 0.0054	rho = 0.2027	*P* = 0.005
GC i	rho = 0.1663	*P* = 0.0121	rho = 0.3636	*P* = 1.6e-08

Intronic GC was calculated only for windows with a minimum of 10 introns present to avoid noise owing to low numbers of sites.

As a consequence of the correlation between recombination and variance in GC, the orthologs from the previously considered high-variance gene set showed higher recombination and variance in recombination in chicken and zebra finch than the low-variance gene set (Wilcoxon test for median rate <2.2e-16; see Table 2). Considering only ‘homogenous’ orthologs yielded similar but modestly weaker correlations (see Table 1), consistent with our above observation that the association between body mass and GC3 extends to these loci. Moreover, intronic GC content and standard deviation also correlated positively with recombination rates (see Table 1).

**Table 2:** **Recombination rates differ between high- and low-variance orthologs**

		**Recombination rate (cM/Mb)**	**Standard deviation**
Chicken	Low-variance	2.035	1.756
	High-variance	4.347	5.609
Zebra finch	Low-variance	0.1035	1.18
	High-variance	3.189	3.599

Chicken and zebra finch recombination rates for 1 Mb windows overlapping the 1,780 orthologs were positively correlated (rho = 0.3846, *P* <2.2e-16), indicating a degree of conservation of recombination rates for our set in line with previously reported estimates [19]. As there is no perfect correspondence between rates, the above correlations are likely to be weaker than if we were able to include only loci whose recombination rates have remained constant across all species. For instance, if a previously highly recombining sequence moved to a region of low recombination and experienced amelioration of GC, the strength of the relationship between chicken chromosomal location and heterogeneity would be reduced. There is indeed evidence that chromosomal inversions are associated with altered recombination rates [26,27].

### Chromosome size predicts GC content and heterogeneity

As interchromosomal rearrangements are rare in birds, we can further employ chromosomal class as a proxy for ‘very broad scale’ recombination rates. Given the requirement for at least one crossover per chromosome [39], small chromosomes have higher recombination rates [19,20,41]. Therefore higher heterogeneity in GC3 compared to larger chromosomes is expected, along with the higher median GC3. Indeed, orthologs on the smaller chicken chromosomes 10-32 showed a higher median standard deviation in GC3 than the larger chromosomes 1-9 (see Table 3). Zebra finch exhibits the same pattern, which is not surprising given the high degree of karyotypic conservation. Similar results were obtained for intronic GC content, with both the chicken and zebra finch median and standard deviation for GC_i_ being higher for orthologs on small chromosomes (Table 3).

**Table 3:** **Median and standard deviation (sd) of GC for orthologs to chicken and zebra finch genes located on large (chromosomes 1-9) and small chromosomes (chromosome 10-) for third codon position and introns**

	**GC small chrom.**	**GC large chrom.**	**Wilcoxon test**	**sd GC small**	**sd GC large**	**Wilcoxon test**
Chicken third sites	0.495	0.458	*P* = 2.0e-11	0.0457	0.0349	*P* <2.2e-16
Chicken intronic	0.493	0.449	*P* = 0.0020	0.0432	0.0353	*P* = 6.7e-07
Zebra finch third sites	0.497	0.457	*P* = 1.5e-13	0.0456	0.0348	*P* <2.2e-16
Zebra finch intronic	0.500	0.447	*P* = 0.00038	0.0418	0.0352	*P* = 4.3e-05

### Avian base composition is not at equilibrium

Previous work on birds has shown that GC content is increasing in a subset of avian lineages [43,47], coinciding with a reinforcement of isochore structure, whereas in other lineages GC has declined. In mammals it was originally assumed that GC was becoming eroded and homogenized based on observations in rodents and primates. A more comprehensive analysis including additional species showed that these examples were in fact exceptions [21]. We therefore ask whether there is evidence for a general trend in GC evolution across all major avian orders. This is typically done by examining the relationship between weak (W: GC → AT) and strong (S: AT → GC) substitutions. Calculating (W → S)/(W → S + S → W) for summed substitution counts obtained by mapping W → S and S → W counts onto the branches of the avian tree using mapNH and a homogenous T92 model yields an approximate GC3* value for each species [66]. This estimate represents the GC3 composition that would be expected for an infinitely long branch.

**Figure 8:**
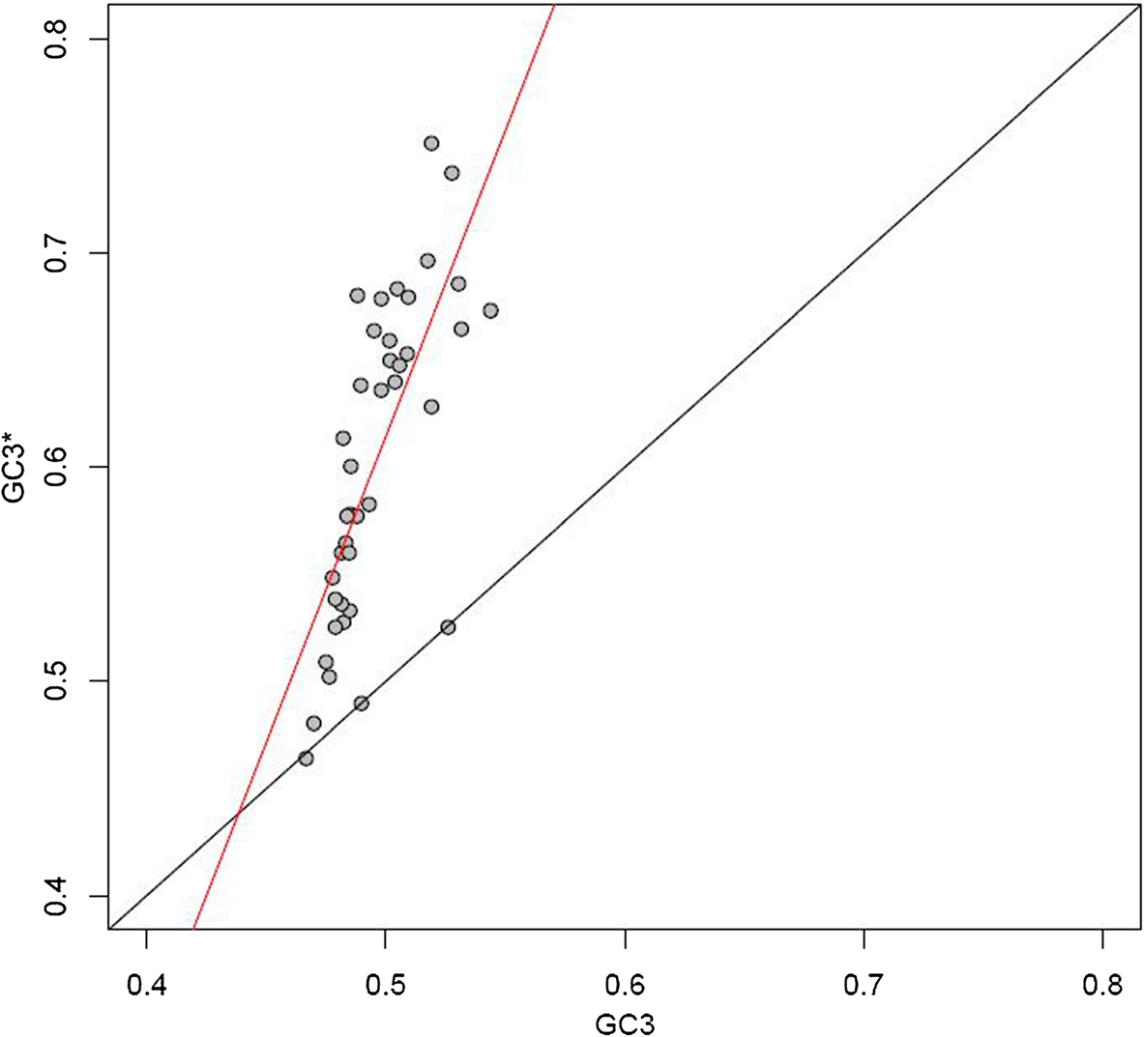
**GC3 is not at equilibrium in the majority of avian species.** For the majority of lineages, GC3* exceeds GC3. As an exception the ostrich, *Struthio camelus*, shows no evidence of increasing GC content (GC3* = 0.4645; GC3 = 0.4666).

At equilibrium, we would expect GC3 and GC3* to be approximately equal. However, in the majority of cases we observed that GC3* was in fact greater than current GC3, with the slope between the two measures being 2.85 (Figure 8), indicating that GC has increased [43]. These equilibrium frequencies should be interpreted with a degree of caution, as they are based on observations of substitutions along branches of finite length and changes in base composition will ultimately lead to a shift in the balance of forces acting on a sequence. The effect of distorted segregation favoring W → S changes on composition ought to become less pronounced as the frequency of unfixed GC variants declines, while the proportion of targets susceptible to C → T transitions increases. Very high GC contents could in principle also be selected against in certain sequence contexts in sufficiently large populations, as GC content is a major predictor of nucleosome occupancy and therefore DNA accessibility (see, for example, [67]). As such, the notion that high N_e_ will accelerate gBGC [23] may not hold in all scenarios. Given that we do not control for the effects of CpG hypermutability on substitutions in our model, it is possible that we overestimate the true equilibrium frequencies here to an extent. Nevertheless, our data provide evidence that, if anything, GC3 has undergone recent increases in most species surveyed. The overall trend for GC3* to be higher than current GC3 is consistent with the idea that, on average, bird body masses have decreased throughout their evolution [7].

Although we cannot conclusively rule out the possibility that the equilibrium GC frequencies we estimated are greater than the frequencies that would be reached in the future given sufficient time and constant population sizes, past work on primates has shown that a model where the efficiency of gBGC depends on recombination and effective population size fits the observed data well [14]. The primate results also suggest that the time required to halve the difference between current and equilibrium composition is of the order of hundreds of millions of years outside of hotspot regions [14]. If we assume a range of 100,000 to 1,000,000 for avian effective population sizes, we can demonstrate using a model similar to that of Duret and Arndt [14] that the number of generations required to halve the difference between GC3* and GC is large - between 124,218,180 and 565,739,002 generations - relative to the time scales we investigate here (see Additional file 5). We might therefore plausibly expect GC3* to exceed current GC3.

## Discussion

Multiple lines of circumstantial evidence described in this study support a role for recombination-associated gBGC in shaping avian base composition. First, lineages with putatively higher N_e_ and shorter generation times show increased GC content at all classes of sites we examined (coding and non-coding), as well as greater heterogeneity in GC3. In addition to life history traits, estimated ancestral population size is a predictor of GC content. These results are consistent with both the effects of a greater number of recombination events occurring per unit time and gBGC being more efficient in large populations [23], and accord with previous reports from mammals [21]. That non-coding sites are also affected argues against the effect being explained by selection on coding sequences. On the other hand, the impact of gBGC is pervasive and appears strong enough to drive some modest differences in amino acid usage between lineages, and extends to loci with low heterogeneity in GC content. The positive correlation in both chicken and zebra finch between recombination and heterogeneity in GC3 as well as median GC3 is consistent with a recombination-associated process increasing GC content. We find that the process appears to be ongoing, with equilibrium GC composition being higher than present composition for the majority of species surveyed. Interestingly, the ostrich, *Struthio camelus*, shows no evidence of increasing GC content (GC3* = 0.4645; GC3 = 0.4666). A reduction in GC relative to the ancestral state has previously been described in emus, *Dromaius novaehollandiae* [47]. Meanwhile, the great tinamou, *Tinamus major*, although closely related to both the emu and ostrich, shows a particularly high GC3*. In fact, ostrich has recently been shown to be outgroup to tinamou and emu [68], further indicating that phylogeny does not account for our observations (Additional file 3). Work on primates shows that the gBGC model fits the observed equilibrium frequencies well [14]. Nevertheless, future work will be required to address the extent to which selection opposing very high GC content or CpG methylation might affect equilibrium composition in coding sequences.

The counterintuitive observation that the range of intronic GC appears to be somewhat more restricted than GC3 (sd = 0.0189 for GC_i_) may be explained by selection on intronic nucleotide composition to facilitate correct intron recognition by the splicing machinery [69,70]. Repeat-masking intronic sequences had a negligible effect on overall intronic GC content (data not shown), indicating that transposable elements do not play an important role in intronic nucleotide composition in birds as opposed to mammals [71]. Alternatively, the weaker effect in introns could be due to a tendency for GC-rich introns to be deleted. Recombination is associated with genome contraction [72] and constraint to maintain intronic sequence is expected to be weaker than for coding sequences with introns having an overall deletion bias [73].

Despite our comprehensive data set across the avian phylogeny and the advantages of the avian system, we acknowledge that some caveats remain. Given that we likely underestimate both generation times and coalescent branch lengths, our reconstructions of population size are imperfect and expected to be larger than the true ancestral population sizes (see Materials and Methods). However, our main objective was to capture the ranks of the ancestral population sizes relative to one another in order to draw conclusions about their relationship with base composition.

At present, the absence of recombination rate data for the majority of our study taxa, as well as the lack of knowledge of historical rates, limits our ability to draw firm conclusions about fine-scale changes in recombination landscapes over time and how this may impact the patterns of base composition we observe. Sex-specific rates might also be expected to give different results in some study organisms. The impact of male recombination on signatures of gBGC is stronger than for female recombination in mammals [14,28,74-76], possibly owing to interactions between replication-associated mutation patterns and crossover [77]. However, no such difference between the effects of male and female recombination on nucleotide composition is reported in chicken [78], although this may be a matter of resolution. Nevertheless, if anything we would expect shifts in the crossover landscape to weaken correlations, making our analysis more conservative.

Future analyses employing both detailed crossover maps and rearrangement data will shed light on whether the reinforcement of GC-rich isochores in birds is indeed related to a connection between the stable avian karyotype and stable recombination landscapes. One explanation that has been proposed for the long-lived recombination hotspots in dog is the loss of *PRDM9* in canids, leading to pronounced signatures consistent with long episodes of gBGC [36]. Like dogs, birds appear to lack a functional copy of the gene [35], implying that karyotypic stability may not be the only possible explanation for what we observe in birds.

Characterizing the strength of gBGC using a model such as that of Lartillot [24] will enable us to gather more information about the longevity of avian recombination hotspots in future studies. In mammals, a mere 20% of the strength of gBGC is accounted for by karyotype and body mass, presumably owing to population size not being perfectly correlated with body mass, and karyotype not accounting for all of the variation in recombination rates [24]. Levels of species inbreeding are also expected to play a role [79]. Therefore, gathering more information about how well body mass explains traces left in the genome by gBGC relative to other proxies such as gene tree discordance or levels of heterozygosity will be important. The increasing availability of polymorphism data will also allow fixation biases to be modeled [25], perhaps providing more direct evidence for gBGC in the future.

At present, we cannot fully distinguish between the relative importance of the generation time and population size effects on the strength of GC-biased gene conversion because species with long generation times tend also to have small populations, and our results suggest that GC has not reached equilibrium in the majority of lineages. As with recombination, N_e_ varies both along the genome and between genomes. Indeed recombination itself modulates N_e_ locally. This is however likely to be of limited importance. Differences in N_e_ between genes along the chromosomes are small in the species thus far surveyed [22] suggesting that the modestly increased N_e_ of highly recombining sequences might affect the efficacy of gBGC less than the overall species-specific N_e_. Whether these predictions also hold for birds, where one might expect to see more pronounced intragenomic variation in N_e_ owing to heterogeneous recombination rates, is as of yet not clear. Moreover, the extent to which selection on synonymous or non-coding sites might modulate the impact of gBGC on composition remains to be explored.

More generally, our results show that the relationship between life history traits and base composition is not limited to mammalian species, as would be expected for a mechanism that is conserved across a wide range of eukaryotic species. Interestingly, the sea lamprey genome, which contains a large number of micro-chromosomes has an extremely high GC3 content, in principle consistent with recombination driving an increase in GC. Surprisingly, despite the connection between the strength of gBGC and chromosome number, no significant correlation between GC and GC3 was reported [80]. Given the abundance of repetitive sequence in the lamprey and the fact that unmasked sequences were assayed, this may however be a method artefact rather than evidence against gBGC as a driver of GC in this particular genome.

## Conclusions

Here, we have demonstrated the pervasive impact of gBGC on avian base composition and shown that life history affects lineage-specific patterns of GC evolution. The observation that a species’ GC content can be predicted from its life history for multiple taxa strengthens the plausibility of gBGC acting as a driver of between-lineage differences in composition in addition to its well-established role as a determinant of within-species variation. Our results are in line with the expectation that a mechanism that is conserved across multiple eukaryotic groups ought to leave similar traces in their genomes, as appears to be the case for mammals and birds.

Our findings are also of broad interest for phylogenetic reconstruction, as there is increasing evidence that base composition can substantially alter the topologies of the trees inferred from different classes of sequence [33,47]. This is further elaborated on in our large-scale phylogenomic analysis of the genome sequences used herein [33]. Beyond model misspecification related to base composition, high recombination could affect phylogenetic inference in several ways, for instance via its association with incomplete lineage sorting or, in lineages with rapid turnover of hotspots, by frequent switches in the substitution regime [81]. Gathering further information on the dynamics of evolution of avian recombination landscapes in the future will shed light on whether the latter mechanism is likely to be of importance in birds. More generally, the impact of a mechanism that drives alleles to fixation in absence of positive selection on lineage-specific substitutions [10] may be of practical importance for the interpretation of evolutionary rates.

## Materials and methods

### Sequence data

This work was a companion study to a recent initiative to resolve the phylogeny of modern birds and coding and intronic sequence alignments were obtained from the Avian Phylogenomics Consortium [33,48], who provide a detailed description of how data were generated. Briefly, this data set comprises 8,295 orthologous protein coding sequences identified by propagating chicken and zebra finch annotations to the remaining species and classifying orthology by combining information from alignment statistics, reciprocal best hits and synteny. Introns for these orthologs were defined by identifying gene-models with conserved exon-intron boundaries. Multiple sequence alignments were generated by running SATé + PRANK followed by SATé + MAFFT on concatenated exon sequences and two rounds of SATé + MAFFT on introns [33].

### Nucleotide composition

GC content was calculated for first, second, and third coding positions, as well as at intronic sites for each species. Only those orthologs present in all species were considered (*n* = 1,780) to ensure comparisons between species were made between comparable coding sequences. Nucleotide content was tallied for all sites at a given position before computing the proportion of GC nucleotides, so that short sequences do not introduce noise. Similarly, for introns only those associated with genes found in the above set of orthologs were considered (*n* = 404). Additionally, nucleotide content was calculated in the above manner for all 8,295 orthologs for c123, c12, c3 and introns.

### Life history traits

Body mass data were extracted from the *CRC Handbook of Avian Body Masses* [82], with only data for unambiguously named tip nodes being used. Where multiple entries for a given species were present, the mean value was calculated. We therefore averaged across males and females where these were not already pooled in the dataset. Data on maximum longevity and age of first female sexual maturity were extracted from build 11 of the AnAge database [83] for each available species.

### Reconstructed ancestral generation times

To obtain N, we required estimates of ancestral generation time. Given the limited availability of generation time data, ancestral age of first female sexual maturity was reconstructed using Coevol [64] on 10 subsets of 10 kb of concatenated sequence drawn randomly from the 1,185 1:1 orthologs. This approach allows ancestral traits to be estimated by combining a Brownian diffusion process and correlations between substitution rates and life history traits. As species d_N_/d_S_ does not correlate with life history traits in the manner expected under nearly neutral theory in birds [84], we employed overall substitution rates for the reconstructions (that is, Coevol was run without the codon model option). The prior for the root was set to 100 My, which is close to the estimated age in the dated tree [33], with a standard deviation of 100 My, and a burn-in of 100 points was used to evaluate the chains. Reconstructed estimates were highly correlated between the 10 concatenated alignments (Additional file 6), indicating that 10 kb of sequence were sufficient to give reproducible results while being less computationally costly. We ran additional Coevol chains with the above parameters allowing for variation in equilibrium GC, which in turn allowed us to estimate GC3* for internal branches and examine the correlations between GC and age of first female sexual maturity through time.

Ancestral generation time g is typically calculated by *g* = *a* + [*s* ⁄ (1 - *s*)] where *s* denotes the expected adult survival rate and *a* is age of first female sexual maturity. As we lack a curated source for *s* for our species, we considered whether approximating ancestral survival by fitting a regression model to data from 271 bird species for which both *g* and *a* are available [85] would improve our estimation of N. However, after performing these calculations (s ⁄ (1 - s)) tended to be inflated in late-reproducing birds with high survival rates, leading to inferred generation times that were greater than maximum longevity. We therefore opted to use age of first female maturity as a proxy for generation time.

### Ancestral population size

Gene tree topologies were compared to the species tree with a double-recursive tree traversal similar to that used in PHYLDOG [86]. Briefly, the nodes of the gene tree (MP-EST in [33]) were first mapped onto nodes of the species tree (TENT ExaML in [33]), and then the number of gene lineages at the beginning and at the end of each branch of the species tree are recorded. These numbers are computed for all gene trees. Then these numbers are used to estimate branch lengths in coalescent units (coalescent units correspond to the number of generations divided by the effective population size along a branch of the species tree) using the following formula, for the branch *i*:

$$ \mathrm{length}\ \mathrm{of}\ \mathrm{branch}\ \mathrm{i} = \log\ \left(\left(n 12+nkk\right)/nkk\right) $$

where *n12* is the number of times one gene was found at the beginning of *branch i*, and two genes were found at the end of *branch i* over all gene trees, and *nkk* is the number of times *k* genes were found at the beginning and the end of *branch i* all gene trees, *k* ≠ *1*. This formula is an approximation based on equation (2) in reference [63], and was found to work well on simulated data (data not shown). Additionally, branch lengths were also calculated with MP-EST version 1.4 [87].

Thus, for a given branch the reconstructed population size is:

$$ \mathrm{N} = \mathrm{internodal}\ \mathrm{time}\ \mathrm{span}/\left(2*\mathrm{coalescent}\ \mathrm{branch}\ \mathrm{length}*\mathrm{reconstructed}\ \mathrm{generation}\ \mathrm{time}\right) $$

where reconstructed generation time is the mean of the values inferred by Coevol for the nodes at either end of the branch. Internodal time spans were obtained from reference [33]. Note that underestimated coalescent branch lengths will inflate estimates of N by decreasing the divisor of the equation. This is expected to be particularly problematic for poorly resolved parts of the tree, where errors in the gene trees are most frequent. Noise due to lack of information may tend to homogenize the frequencies of the gene trees, leading branch lengths to be underestimated. High levels of recombination in avian genomes are expected to exacerbate this problem.

### Time corrected GC3 conservation

Following the method of Romiguier *et al.* [61], we computed a time corrected index of GC3-conservation for 19 independent pairs of modern bird species. This index is *γ* = -t/log(*τ*), where *t* is the divergence time of the species pair and *τ* the Kendall’s correlation coefficient of gene GC3 in species 1 vs species 2 (830 ortholog families with the highest GC3 variance).

In order to have comparable body-mass in each pair, we chose species that maximized the number of closely related pairs (Additional file 7). We excluded the two Paleognathes (ostrich and great tinamou) because of their extreme contrast in body mass. Among the Neognathae, we chose the *Haliaeetus albicilla* (white-tailed eagle)/*Cathartes aura* (turkey vulture) pair over the intra-genus *Haliaeetus albicilla*/*Haliaeetus leucocephalus* pair (bald eagle) because of the extremely short divergence time of the latter. These 19 time-corrected measures of GC3-conservation were then correlated with the mean body mass of the corresponding species pair.

### Recombination rates

Recombination rates for 1 Mb windows were obtained for chicken [20] and zebra finch [19]. Orthologs were mapped to their corresponding 1 Mb windows and GC for the sequences of interest was then computed for each of these windows.

### Statistical analyses

All statistics were calculated in R.

### Data availability

The genome sequences used in this study are available from GigaDB [88].

## Additional files

Additional file 1:
**List of species abbreviations used in figures.**
Additional file 2:
**Relationships between amino acid usage and body mass.**
Additional file 3:
**Phylogenetic independent contrasts.**
Additional file 4:
**Correlations though time between dS, age of female sexual maturity, and GC3.**
Additional file 5:
**Estimates of equilibrium process half times.**
Additional file 6:
**Correlations between Coevol reconstructed traits for 10 concatenated alignments.**
Additional file 7:
**Species pairs used for GC3 conservation analysis.**

## Abbreviations

d_N_/d_S_: ratio of synonymous to non-synonymous substitutions
gBGC: GC-biased gene conversion
GC3: GC content at third codon positions
GC3*: equilibrium GC at third codon positions
LHTs: Life history traits
Mb: Megabase pair
N_e_: Effective population size
S: strong substitution
W: weak substitution

## References

[1] Wilson Sayres MA, Venditti C, Pagel M, Makova KD (2011). Do variations in substitution rates and male mutation bias correlate with life-history traits? A study of 32 mammalian genomes. Evolution.

[2] Lartillot N, Delsuc F (2012). Joint reconstruction of divergence times and life-history evolution in placental mammals using a phylogenetic covariance model. Evolution.

[3] Thomas JA, Welch JJ, Lanfear R, Bromham L (2010). A generation time effect on the rate of molecular evolution in invertebrates. Mol Biol Evol.

[4] Bromham L (2011). The genome as a life-history character: why rate of molecular evolution varies between mammal species. Philos Trans R Soc Lond B Biol Sci.

[5] Lanfear R, Kokko H, Eyre-Walker A (2014). Population size and the rate of evolution. Trends Ecol Evol.

[6] Akashi H, Osada N, Ohta T (2012). Weak selection and protein evolution. Genetics.

[7] Nabholz B, Uwimana N, Lartillot N (2013). Reconstructing the phylogenetic history of long-term effective population size and life-history traits using patterns of amino acid replacement in mitochondrial genomes of mammals and birds. Genome Biol Evol.

[8] Mancera E, Bourgon R, Brozzi A, Huber W, Steinmetz LM (2008). High-resolution mapping of meiotic crossovers and non-crossovers in yeast. Nature.

[9] Lesecque Y, Mouchiroud D, Duret L (2013). GC-biased gene conversion in yeast Is specifically associated with crossovers: Molecular mechanisms and evolutionary significance. Mol Biol Evol.

[10] Galtier N, Duret L (2007). Adaptation or biased gene conversion? Extending the null hypothesis of molecular evolution. Trends Genet.

[11] Galtier N, Piganeau G, Mouchiroud D, Duret L (2001). GC-content evolution in mammalian genomes: the biased gene conversion hypothesis. Genetics.

[12] Webster MT, Hurst LD (2012). Direct and indirect consequences of meiotic recombination: implications for genome evolution. Trends Genet.

[13] Muyle A, Serres-Giardi L, Ressayre A, Escobar J, Glémin S (2011). GC-biased gene conversion and selection affect GC content in the *Oryza genus* (rice). Mol Biol Evol.

[14] Duret L, Arndt PF (2008). The impact of recombination on nucleotide substitutions in the human genome. PLoS Genet.

[15] Duret L, Galtier N (2009). Biased gene conversion and the evolution of mammalian genomic landscapes. Annu Rev Genomics Hum Genet.

[16] Pessia E, Popa A, Mousset S, Rezvoy C, Duret L, Marais GAB (2012). Evidence for widespread GC-biased gene conversion in eukaryotes. Genome Biol Evol.

[17] Birdsell JA (2002). Integrating genomics, bioinformatics, and classical genetics to study the effects of recombination on genome evolution. Mol Biol Evol.

[18] Spencer CCA, Deloukas P, Hunt S, Mullikin J, Myers S, Silverman B, Donnelly P, Bentley D, McVean G (2006). The influence of recombination on human genetic diversity. PLoS Genet.

[19] Backström N, Forstmeier W, Schielzeth H, Mellenius H, Nam K, Bolund E, Webster MT, Ost T, Schneider M, Kempenaers B, Ellegren H (2010). The recombination landscape of the zebra finch *Taeniopygia guttata* genome. Genome Res.

[20] Groenen MAM, Wahlberg P, Foglio M, Cheng HH, Megens H-j, Crooijmans RPM, Besnier F, Lathrop M, Muir WM, Wong GK-S, Gut I, Andersson L (2009). A high-density SNP-based linkage map of the chicken genome reveals sequence features correlated with recombination rate. Genome Res.

[21] Romiguier J, Ranwez V, Douzery EJP, Galtier N (2010). Contrasting GC-content dynamics across 33 mammalian genomes: relationship with life-history traits and chromosome sizes. Genome Res.

[22] Gossmann TI, Woolfit M, Eyre-Walker A (2011). Quantifying the variation in the effective population size within a genome. Genetics.

[23] Nagylaki T (1983). Evolution of a finite population under gene conversion. Proc Natl Acad Sci U S A.

[24] Lartillot N (2013). Phylogenetic patterns of GC-biased gene conversion in placental mammals and the evolutionary dynamics of recombination landscapes. Mol Biol Evol.

[25] de Maio N, Schlötterer C, Kosiol C (2013). Linking great Apes genome evolution across time scales using polymorphism-aware phylogenetic models. Mol Biol Evol.

[26] Auton A, Fledel-Alon A, Pfeifer S, Venn O, Ségurel L, Street T, Leffler EM, Bowden R, Aneas I, Broxholme J, Humburg P, Iqbal Z, Lunter G, Maller J, Hernandez RD, Melton C, Venkat A, Nobrega MA, Bontrop R, Myers S, Donnelly P, Przeworski M, McVean G (2012). A fine-scale chimpanzee genetic map from population sequencing. Science.

[27] Farré M, Micheletti D, Ruiz-Herrera A (2013). Recombination rates and genomic shuffling in human and chimpanzee–a new twist in the chromosomal speciation theory. Mol Biol Evol.

[28] Clément Y, Arndt PF (2011). Substitution patterns are under different influences in primates and rodents. Genome Biol Evol.

[29] Ellegren H (2010). Evolutionary stasis: the stable chromosomes of birds. Trends Ecol Evol.

[30] Shetty S, Griffin DK, Graves JA (1999). Comparative painting reveals strong chromosome homology over 80 million years of bird evolution. Chromosome Res.

[31] Derjusheva S, Kurganova A, Habermann F, Gaginskaya E (2004). High chromosome conservation detected by comparative chromosome painting in chicken, pigeon and passerine birds. Chromosome Res.

[32] Guttenbach M, Nanda I, Feichtinger W, Masabanda JS, Griffin DK, Schmid M (2003). Comparative chromosome painting of chicken autosomal paints 1–9 in nine different bird species. Cytogenet Genome Res.

[33] Jarvis ED, Mirarab S, Aberer AJ, Li B, Houde P, Li C, Ho SYW, Faircloth BC, Nabholz B, Howard JT, Suh A, Weber CC, da Fonseca RR, Li J, Zhang F, Li H, Zhou L, Narula N, Liu L, Ganapathy G, Boussau B, Bayzid MS, Zavidovych V, Subramanian S, Gabaldón T, Capella-Gutiérrez S, Huerta-Cepas J, Rekepalli B, Munch K, Schierup M (2014). Whole-genome analyses resolve early branches in the tree of life of modern birds. Science.

[34] Warren WC, Clayton DF, Ellegren H, Arnold AP, Hillier LW, Künstner A (2010). The genome of a songbird. Nature.

[35] Oliver PL, Goodstadt L, Bayes JJ, Birtle Z, Roach KC, Phadnis N, Beatson S, Lunter G, Malik HS, Ponting CP (2009). Accelerated evolution of the *Prdm9* speciation gene across diverse metazoan taxa. PLoS Genet.

[36] Axelsson E, Webster MT, Ratnakumar A, Ponting CP, Lindblad-Toh K (2012). Death of *PRDM9* coincides with stabilization of the recombination landscape in the dog genome. Genome Res.

[37] Lesecque Y, Glémin S, Lartillot N, Mouchiroud D, Duret L (2014). The Red Queen model of recombination hotspots evolution in the light of archaic and modern human genomes. PLoS Genet.

[38] Mugal CF, Arndt PF, Ellegren H (2013). Twisted signatures of GC-biased gene conversion embedded in an evolutionary stable karyotype. Mol Biol Evol.

[39] Martini E, Diaz RL, Hunter N, Keeney S (2006). Crossover homeostasis in yeast meiosis. Cell.

[40] McQueen HA, Siriaco G, Bird AP, Mcqueen HA (1998). Chicken microchromosomes are hyperacetylated, early replicating, and gene rich. Genome Res.

[41] ICGSC (2004). Sequence and comparative analysis of the chicken genome provide unique perspectives on vertebrate evolution. Nature.

[42] Axelsson E, Webster M, Smith N, Burt D, Ellegren H (2005). Comparison of the chicken and turkey genomes reveals a higher rate of nucleotide divergence on microchromosomes than macrochromosomes. Genome Res.

[43] Webster MT, Axelsson E, Ellegren H (2006). Strong regional biases in nucleotide substitution in the chicken genome. Mol Biol Evol.

[44] Duret L, Semon M, Mouchiroud D, Galtier N (2002). Vanishing GC-rich isochores in mammalian genomes. Genetics.

[45] Belle EMS, Duret L, Galtier N, Eyre-Walker A (2004). The decline of isochores in mammals: an assessment of the GC content variation along the mammalian phylogeny. J Mol Evol.

[46] Smith NGC, Eyre-Walker A (2002). The compositional evolution of the murid genome. J Mol Evol.

[47] Nabholz B, Künstner A, Wang R, Jarvis ED, Ellegren H (2011). Dynamic evolution of base composition: causes and consequences in avian phylogenomics. Mol Biol Evol.

[48] Zhang G, Li C, Li Q, Li B, Larkin DM, Lee C, Storz JF, Antunes A, Greenwold MJ, Meredith RW, Odeen A, Cui J, Zhou Q, Xu L, Pan H, Wang Z, Jin L, Zhang P, Hu H, Yang W, Hu J, Xiao J, Yang Z, Liu Y, Xie Q, Yu H, Lian J, Wen P, Zhang F, Li H (2014). Comprehensive avian phylogenomic analyses reveal novel and fundamental insights on genomic and phenotypic complexities of bird evolution. Science.

[49] dos Reis M, Wernisch L (2009). Estimating translational selection in eukaryotic genomes. Mol Biol Evol.

[50] Künstner A, Nabholz B, Ellegren H (2011). Significant selective constraint at 4-fold degenerate sites in the avian genome and its consequence for detection of positive selection. Genome Biol Evol.

[51] Doherty A, McInerney JO (2013). Translational selection frequently overcomes genetic drift in shaping synonymous codon usage patterns in vertebrates. Mol Biol Evol.

[52] Urrutia AO, Hurst LD (2001). Codon usage bias covaries with expression breadth and the rate of synonymous evolution in humans, but this is not evidence for selection. Genetics.

[53] Plotkin JB, Kudla G (2011). Synonymous but not the same: the causes and consequences of codon bias. Nat Rev Genet.

[54] Chamary J-V, Parmley JL, Hurst LD (2006). Hearing silence: non-neutral evolution at synonymous sites in mammals. Nat Rev Genet.

[55] Piganeau G, Mouchiroud D, Duret L, Gautier C (2002). Expected relationship between the silent substitution rate and the GC content: implications for the evolution of isochores. J Mol Evol.

[56] Park C, Chen X, Yang J-R, Zhang J (2013). Differential requirements for mRNA folding partially explain why highly expressed proteins evolve slowly. Proc Natl Acad Sci U S A.

[57] Zur H, Tuller T (2012). Strong association between mRNA folding strength and protein abundance in S. cerevisiae. EMBO Rep.

[58] Galtier N, Duret L, Glémin S, Ranwez V (2009). GC-biased gene conversion promotes the fixation of deleterious amino acid changes in primates. Trends Genet.

[59] Warnecke T, Weber CC, Hurst LD (2009). Why there is more to protein evolution than protein function: splicing, nucleosomes and dual-coding sequence. Biochem Soc Trans.

[60] Galtier N, Gouy M (1998). Inferring pattern and process: maximum-likelihood implementation of a nonhomogeneous model of DNA sequence evolution for phylogenetic analysis. Eevolution.

[61] Romiguier J, Ranwez V, Douzery EJP, Galtier N (2013). Genomic evidence for large, long-lived ancestors to placental mammals. Mol Biol Evol.

[62] Nee S, Read A, Greenwood J, Harvey P (1991). The relationship between abundance and body size in British birds. Nature.

[63] Rosenberg NA (2002). The probability of topological concordance of gene trees and species trees. Theor Popul Biol.

[64] Lartillot N, Poujol R (2011). A phylogenetic model for investigating correlated evolution of substitution rates and continuous phenotypic characters. Mol Biol Evol.

[65] Duret L, Eyre-Walker A, Galtier N (2006). A new perspective on isochore evolution. Gene.

[66] Romiguier J, Figuet E, Galtier N, Douzery EJP, Boussau B, Dutheil JY, Ranwez V (2012). Fast and robust characterization of time-heterogeneous sequence evolutionary processes using substitution mapping. PLoS One.

[67] Warnecke T, Batada NN, Hurst LD (2008). The impact of the nucleosome code on protein-coding sequence evolution in yeast. PLoS Genet.

[68] Haddrath O, Baker AJ (2012). Multiple nuclear genes and retroposons support vicariance and dispersal of the palaeognaths, and an Early Cretaceous origin of modern birds. Proc Biol Sci.

[69] Amit M, Donyo M, Hollander D, Goren A, Kim E, Gelfman S, Lev-Maor G, Burstein D, Schwartz S, Postolsky B, Pupko T, Ast G (2012). Differential GC content between exons and introns establishes distinct strategies of splice-site recognition. Cell Rep.

[70] Gelfman S, Cohen N, Yearim A, Ast G (2013). DNA-methylation effect on cotranscriptional splicing is dependent on GC architecture of the exon-intron structure. Genome Res.

[71] Duret L, Hurst LD (2001). The elevated GC content at exonic third sites is not evidence against neutralist models of isochore evolution. Mol Biol Evol.

[72] Nam K, Ellegren H (2012). Recombination drives vertebrate genome contraction. PLoS Genet.

[73] Johnson KP (2004). Deletion bias in avian introns over evolutionary timescales. Mol Biol Evol.

[74] Webster MT, Smith NGC, Hultin-Rosenberg L, Arndt PF, Ellegren H (2005). Male-driven biased gene conversion governs the evolution of base composition in human alu repeats. Mol Biol Evol.

[75] Dreszer TR, Wall GD, Haussler D, Pollard KS (2007). Biased clustered substitutions in the human genome: the footprints of male-driven biased gene conversion. Genome Res.

[76] Berglund J, Pollard KS, Webster MT (2009). Hotspots of biased nucleotide substitutions in human genes. PLoS Biol.

[77] Pink CJ, Hurst LD (2011). Late replicating domains are highly recombining in females but have low male recombination rates: implications for isochore evolution. PLoS One.

[78] Popa A, Samollow P, Gautier C, Mouchiroud D (2012). The sex-specific impact of meiotic recombination on nucleotide composition. Genome Biol Evol.

[79] Glémin S (2011). Surprising fitness consequences of GC-biased gene conversion. II Heterosis. Genetics.

[80] Smith JJ, Kuraku S, Holt C, Sauka-Spengler T, Jiang N, Campbell MS, Yandell MD, Manousaki T, Meyer A, Bloom OE, Morgan JR, Buxbaum JD, Sachidanandam R, Sims C, Garruss AS, Cook M, Krumlauf R, Wiedemann LM, Sower SA, Decatur WA, Hall JA, Amemiya CT, Saha NR, Buckley KM, Rast JP, Das S, Hirano M, McCurley N, Guo P, Rohner N (2013). Sequencing of the sea lamprey (*Petromyzon marinus*) genome provides insights into vertebrate evolution. Nat Genet.

[81] Romiguier J, Ranwez V, Delsuc F, Galtier N, Douzery EJP (2013). Less is more in mammalian phylogenomics: AT-rich genes minimize tree conflicts and unravel the root of placental mammals. Mol Biol Evol.

[82] Dunning JBJ (2007). CRC Handbook of Avian Body Masses.

[83] de Magalhães JP, Costa J (2009). A database of vertebrate longevity records and their relation to other life-history traits. J Evol Biol.

[84] Weber CC, Nabholz B, Romiguier J, Ellegren H (2014). Kr/Kc but not dN/dS correlates positively with body mass in birds, raising implications for inferring lineage-specific selection. Genome Biol.

[85] Møller AP (2006). Sociality, age at first reproduction and senescence: comparative analyses of birds. J Evol Biol.

[86] Boussau B, Szöllosi GJ, Duret L, Gouy M, Tannier E, Daubin V (2013). Genome-scale coestimation of species and gene trees. Genome Res.

[87] Liu L, Yu L, Edwards SV (2010). A maximum pseudo-likelihood approach for estimating species trees under the coalescent model. BMC Evol Biol.

[88] Zhang G, Li B, Li C, Gilbert MTP, Jarvis ED, The Avian Phylogenomics Consortium, Wang J: **The avian phylogenomics project data.** *GigaScience Database* 2014, http://dx.doi.org/10.5524/101000 10.1186/2047-217X-3-26 PMC4322804 25671091

