# Supplementary material for: Evidence for GC-biased gene conversion as a driver of between-lineage differences in avian base composition
Source: Genome Biol. 2014 Dec 11;15(12):549. doi: 10.1186/s13059-014-0549-1 (PMC4290106; doi:10.1186/s13059-014-0549-1)
Supplement: Additional file 1: — List of species abbreviations used in figures. [file 13059_2014_549_MOESM1_ESM.pdf]

EVIDENCE FOR GC-BIASED GENE CONVERSION AS A DRIVER OF BETWEEN-LINEAGE DIFFERENCES IN AVIAN BASE COMPOSITION - WEBER ET AL. (2014)

Supplementary file 1

Code	Scientific name	Trivial name
ACACH	*Acanthisitta chloris*	Rifleman
ANAPL	*Anas platyrhynchos*	Mallard (domestic)
ANOCA	*Anolis carolinensis*	Green lizard
APAVI	*Apaloderma vittatum*	Bar-tailed Trogon
APTFO	*Aptenodytes forsteri*	Emperor Penguin
BALRE	*Balearica regulorum*	Grey Crowned Crane
BUCRH	*Buceros rhinoceros*	Rhinoceros Hornbill
CALAN	*Calypte anna*	Anna's Hummingbird
CAPCA	*Antrostomus carolinensis*	Chuck-will's-widow (Nightjar)
CARCR	*Cariama cristata*	Red-legged Seriema
CATAU	*Cathartes aura*	Turkey Vulture
CHAPE	*Chaetura pelagica*	Chimney Swift
CHAVO	*Charadrius vociferus*	Killdeer
CHLUN	*Chlamydotis macqueenii*	Macqueen's Bustard
COLLI	*Columba livia*	Rock Pigeon (domestic)
COLST	*Colius striatus*	Speckled Mousebird
CORBR	*Corvus brachyrhynchos*	American Crow
CUCCA	*Cuculus canorus*	Common Cuckoo
EGRGA	*Egretta garzetta*	Little Egret
EURHE	*Eurypyga helias*	Sunbittern
FALPE	*Falco peregrinus*	Peregrine Falcon
FULGL	*Fulmarus glacialis*	Northern Fulmar
GALGA	*Gallus gallus*	Red Junglefowl (Chicken)
GAVST	*Gavia stellata*	Red-throated Loon
GEOFO	*Geospiza fortis*	Medium Ground-finch
HALAL	*Haliaeetus albicilla*	White-tailed Eagle
HALLE	*Haliaeetus leucocephalus*	Bald eagle
LEPDI	*Leptosomus discolor*	Cuckoo Roller
MANVI	*Manacus vitellinus*	Golden-collared Manakin
MELGA	*Meleagris gallopavo*	Turkey
MELUN	*Melopsittacus undulatus*	Budgerigar
MERNU	*Merops nubicus*	Carmine Bee-eater
MESUN	*Mesitornis unicolor*	Brown Mesite
NESNO	*Nestor notabilis*	Kea
NIPNI	*Nipponia nippon*	Crested Ibis
OPHHO	*Opisthocomus hoazin*	Hoatzin
PELCR	*Pelecanus crispus*	Dalmatian Pelican
PHACA	*Phalacrocorax carbo*	Cormorant
PHALE	*Phaethon lepturus*	White-tailed Tropicbird
PHORU	*Phoenicopterus ruber*	American Flamingo

PICPU	*Picoides pubescens*	Downy Woodpecker
PODCR	*Podiceps cristatus*	Great Crested Grebe
PTEGU	*Pterocles gutturalis*	Yellow-throated Sandgrouse
PYGAD	*Pygoscelis adeliae*	Adelie Penguin
STRCA	*Struthio camelus*	Ostrich
TAEGU	*Taeniopygia guttata*	Zebra Finch
TAUER	*Tauraco erythrolophus*	Red-crested Turaco
TINMA	*Tinamus major*	Great Tinamou
TYTAL	*Tyto alba*	Barn Owl
