# Supplementary material for: Evidence for GC-biased gene conversion as a driver of between-lineage differences in avian base composition
Source: Genome Biol. 2014 Dec 11;15(12):549. doi: 10.1186/s13059-014-0549-1 (PMC4290106; doi:10.1186/s13059-014-0549-1)
Supplement: Additional file 2: — Relationships between amino acid usage and body mass. [file 13059_2014_549_MOESM2_ESM.pdf]

EVIDENCE FOR GC-BIASED GENE CONVERSION AS A DRIVER OF BETWEEN-LINEAGE DIFFERENCES IN AVIAN BASE COMPOSITION - WEBER ET AL. (2014)

Supplementary file 2

Given the pervasive effects of GC-biased gene converion and correlation between GC content and 2nd codon positions, we might expect that life history is also associated with differences in amino acid usage between species due to the structure of the genetic code. This appears indeed to be the case for a subset of amino acids. Figures represent amino acid usage in percent plotted against log(body mass) in grams, with amino acids labelled in one-letter notation. Rho denotes Spearman's rank correlation coefficient.

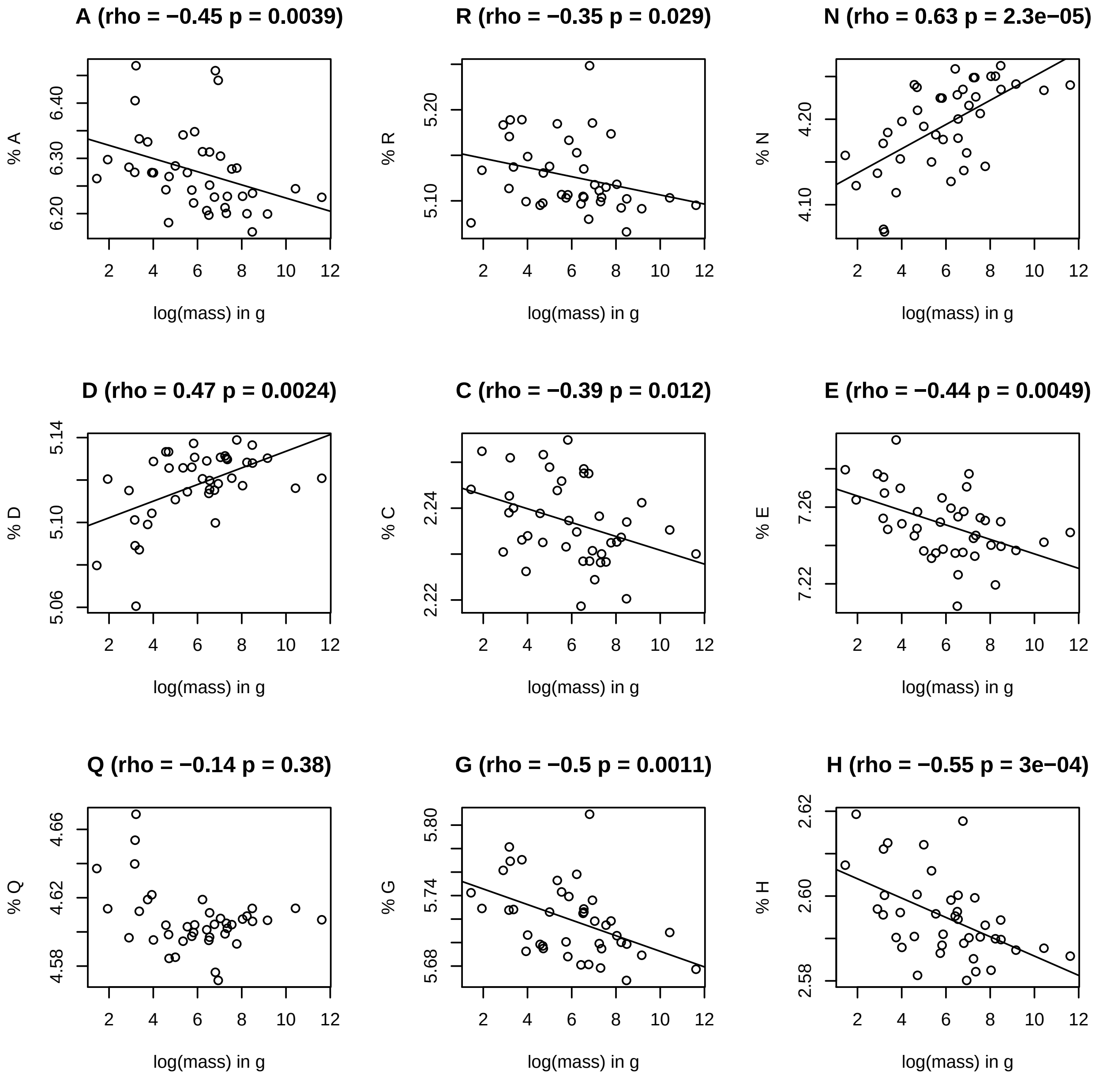
A (rho = −0.45 p = 0.0039)
R (rho = −0.35 p = 0.029)
N (rho = 0.63 p = 2.3e−05)
D (rho = 0.47 p = 0.0024)
C (rho = −0.39 p = 0.012)
E (rho = −0.44 p = 0.0049)
Q (rho = −0.14 p = 0.38)
G (rho = −0.5 p = 0.0011)
H (rho = −0.55 p = 3e−04)
% A
% R
% N
% D
% C
% E
% Q
% G
% H
log(mass) in g

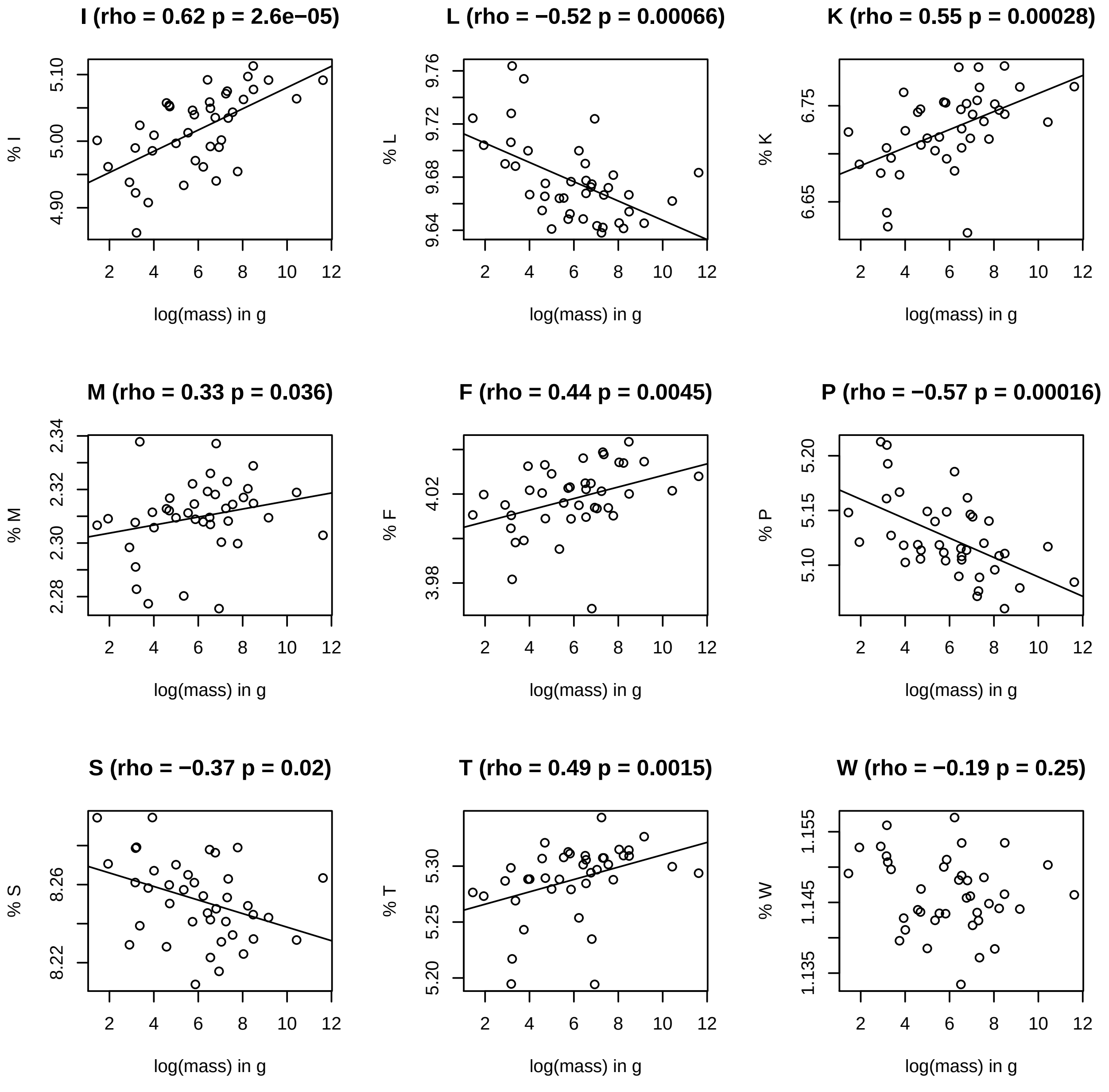
I (rho = 0.62 p = 2.6e−05)
L (rho = −0.52 p = 0.00066)
K (rho = 0.55 p = 0.00028)
M (rho = 0.33 p = 0.036)
F (rho = 0.44 p = 0.0045)
P (rho = −0.57 p = 0.00016)
S (rho = −0.37 p = 0.02)
T (rho = 0.49 p = 0.0015)
W (rho = −0.19 p = 0.25)
% I
% L
% K
% M
% F
% P
% S
% T
% W
log(mass) in g

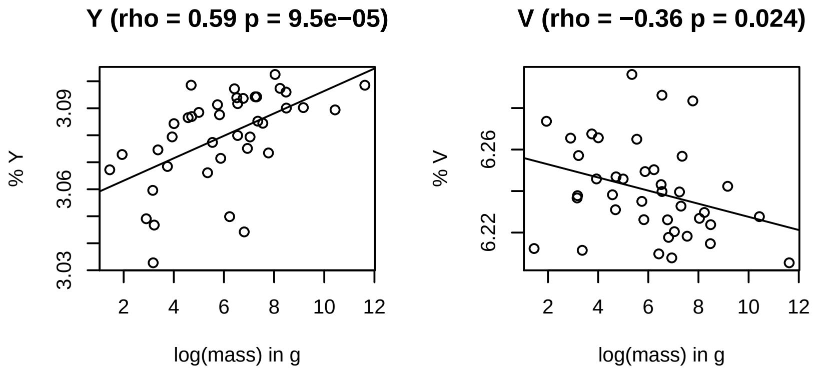
Y (rho = 0.59 p = 9.5e−05)
% Y
3.03
3.06
3.09
2
4
6
8
10
12
log(mass) in g
V (rho = −0.36 p = 0.024)
% V
6.22
6.26
2
4
6
8
10
12
log(mass) in g
