# Supplementary material for: Evidence for GC-biased gene conversion as a driver of between-lineage differences in avian base composition
Source: Genome Biol. 2014 Dec 11;15(12):549. doi: 10.1186/s13059-014-0549-1 (PMC4290106; doi:10.1186/s13059-014-0549-1)
Supplement: Additional file 3: — Phylogenetic independent contrasts. [file 13059_2014_549_MOESM3_ESM.pdf]

Supplementary file 4

Correlations for 1780 orthologs and their introns are largely robust to control for phylogenetic inertia using a variety of trees obtained from Jarvis et al. (2014). The table below shows correlations between GC at different codon positions as well as intronic GC (GCi) and body mass. The first row represents the uncorrected Spearman's rank correlations and subsequent rows show the correlations after applying PIC (part of the Ape package in R). The 3rd codon position becomes borderline significant after control for the tree based on codon 3. However, this is expected, as the topology of this tree is predicted to be strongly affected by GC3. Thus, using this tree for phylogenetic control of the correlation between GC3 and body mass controls in part for GC3 itself. The already weak intronic correlation becomes non-significant in two cases, but actually becomes stronger in two others, perhaps consistent with noisy intronic GC content.

Spearman's rho	GC all coding	GC1	GC2	GC3	GCi
raw correlation	-0.5869<br>p = 6.1e-05	-0.5631<br>p = 0.0001	-0.5639<br>p = 0.0001	-0.5866<br>p = 6.2e-05	-0.4411<br>p = 0.0038
DNA c12 part consensus	-0.4732<br>p = 0.002	-0.4397<br>p = 0.0043	-0.4742<br>p = 0.0019	-0.4777<br>p = 0.0018	-0.4848<br>p = 0.001
DNA c123 part best	-0.3331<br>p = 0.0339	-0.3314<br>p = 0.0349	-0.3535<br>p = 0.0239	-0.3505<br>p = 0.0252	-0.2223<br>p = 0.162
Codon 3 MRE	-0.2901<br>p = 0.0661	-0.3232<br>p = 0.0398	-0.3838<br>p = 0.0138	-0.272<br>p = 0.0856	-0.2847<br>p = 0.0715
Whole genome indel best	-0.4382<br>p = 0.0045	-0.4134<br>p = 0.0076	-0.4378<br>p = 0.0045	-0.4303<br>p = 0.0053	-0.3233<br>p = 0.0397
Intron part MRE consensus	-0.4843<br>p = 0.0015	-0.4519<br>p = 0.0033	-0.4847<br>p = 0.0015	-0.4749<br>p = 0.0019	-0.4817<br>p = 0.0016
Amino acid Fast Tree	-0.3699<br>p = 0.0178	-0.3655<br>p = 0.0193	-0.4186<br>p = 0.0068	-0.3535<br>p = 0.0239	-0.3089<br>p = 0.0499
