# Supplementary material for: Evidence for GC-biased gene conversion as a driver of between-lineage differences in avian base composition
Source: Genome Biol. 2014 Dec 11;15(12):549. doi: 10.1186/s13059-014-0549-1 (PMC4290106; doi:10.1186/s13059-014-0549-1)
Supplement: Additional file 4: — Correlations though time between dS, age of female sexual maturity, and GC3. [file 13059_2014_549_MOESM4_ESM.pdf]

Supplementary file 4

Alignment	dS vs maturity		GC3 vs maturity	
	correlation	pp	correlation	pp
1	-0.824	0	-0.14	0.28
2	-0.772	0	-0.151	0.27
3	-0.78	0	-0.418	0.069
4	-0.854	0	-0.697	0.0004
5	-0.777	0	-0.588	0.0094
6	-0.757	0	-0.29	0.11
7	-0.962	0	-0.806	0.0054
8	-0.79	0	-0.434	0.036
9	-0.721	0.0004	-0.641	0.005
10	-0.811	0	-0.52	0.042

Results from 10 Coevol chains (see Materials and Methods) show negative correlations through time between age of 1st female sexual maturity (labelled "maturity") and dS, which was used to model trait evolution along the tree. They also show a negative relationship between GC3 and age of 1st female sexual maturity through time. Although not all alignments reached low posterior probabilities, this is consistent with our observation that small-bodied birds with short generation times and large populations tend to have higher GC3.
