# Supplementary material for: Evidence for GC-biased gene conversion as a driver of between-lineage differences in avian base composition
Source: Genome Biol. 2014 Dec 11;15(12):549. doi: 10.1186/s13059-014-0549-1 (PMC4290106; doi:10.1186/s13059-014-0549-1)

Supplementary file 5

The notion that the number of meioses per yer could influence a species' current GC content hinges on the assumption that equilibrium has yet to be reached. As we demonstrate in Figure 8, this appears to be the case in the majority of species. One might ask whether this is surprising given the number of generations that have passed since the radiation of modern birds. In primates, it has previously been demonstrated that the rate of evolution of base composition, as measured by the rate at which the difference between GC and equilibrium GC is halved (the process half time), is slow: Hundreds of millions of years are required to halve the difference in composition outside of recombination hotspots [14].

To estimate the rate at which base composition evolves in birds, we applied the method of Duret and Arndt (2008) [14] with adjusted parameters. Reports of bird effective population sizes often vary between around 100,000 and 1 million. The gBGC coefficient s has been estimated to be $1e-6$ in chicken [38], while the mutation rate is of the order of $1.5e-9$ [42] and the average bird recombination rate is around 3cM/Mb [41]. We assumed the relative rates of weak to strong and strong to weak mutation to be similar to patterns observed in mammals [14]. As N_e has a strong impact on the inferred process half time (see Figure) we might examine a range of effective population sizes.

If we for instance consider an effective population size of 100,000, we obtain a process half time of 565,739,002 generations. Meanwhile, an effective population of 1,000,000 gives a half time of 124,218,180 generations. Assuming avian generation times of 2-5 years and modern birds having radiated about 100 Mya [33] (i.e. 20M-50M generations ago), these time scales are too short to have reduced the difference between ancestral and equilibrium compositions by half. It is therefore plausible that our observation that GC3 is smaller than GC3* for the majority of species is explained by the fact that the rate of compositional evolution is slow and insufficient time has passed for equilibrium to have been reached.

Figure S5: The rate at which the difference between GC3 and GC3* halves, $t_{1/2}$ depends on N_e. The blue lines delineate the range from 100,000 to 1,000,000. The grey lines indicate 100M, 50M and 20M generations, respectively.

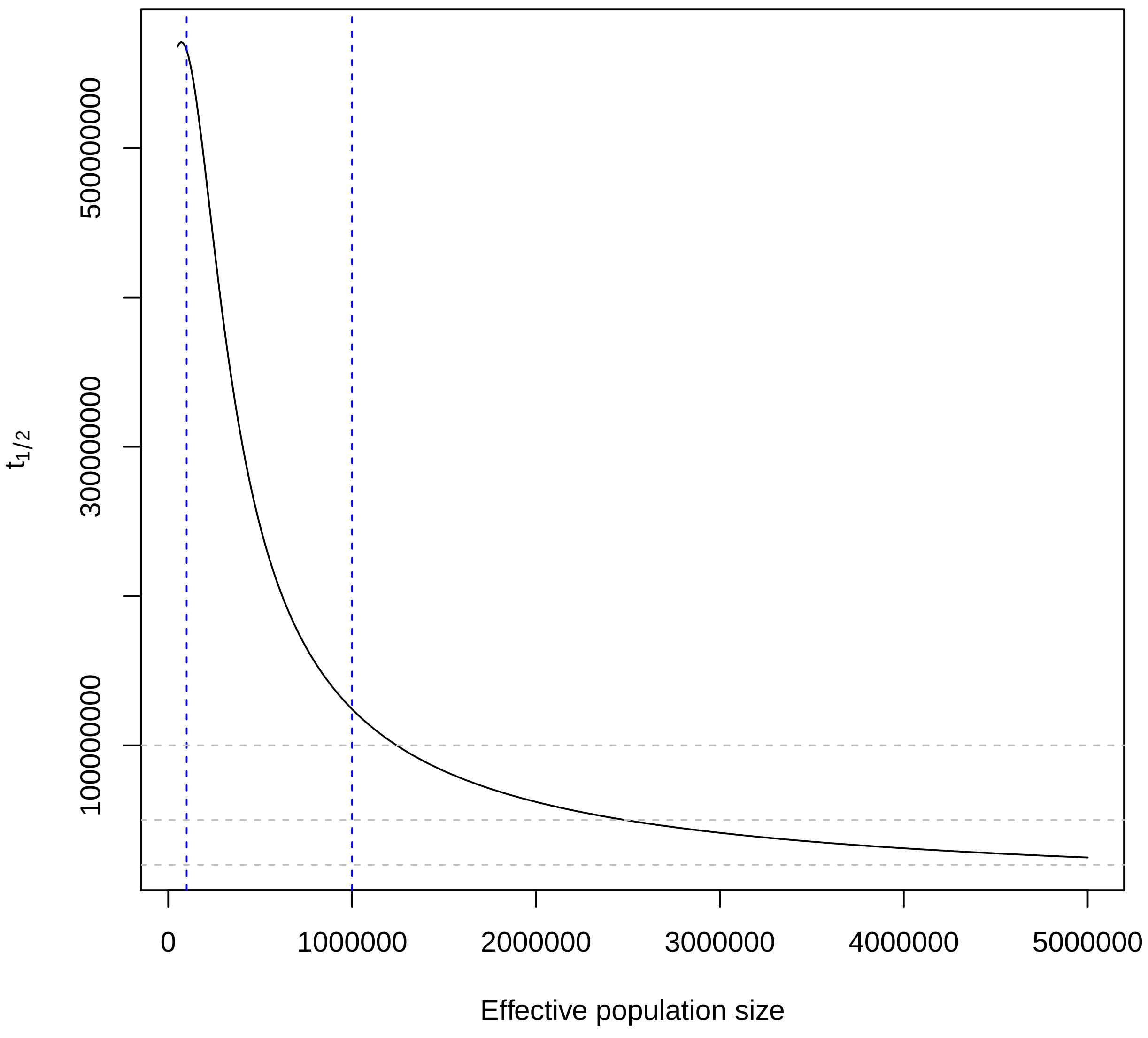

Supplement: Additional file 5: — Estimates of equilibrium process half times. [file 13059_2014_549_MOESM5_ESM.pdf]
