# Supplementary material for: Evidence for GC-biased gene conversion as a driver of between-lineage differences in avian base composition
Source: Genome Biol. 2014 Dec 11;15(12):549. doi: 10.1186/s13059-014-0549-1 (PMC4290106; doi:10.1186/s13059-014-0549-1)
Supplement: Additional file 6: — Correlations between Coevol reconstructed traits for 10 concatenated alignments. [file 13059_2014_549_MOESM6_ESM.pdf]

EVIDENCE FOR GC-BIASED GENE CONVERSION AS A DRIVER OF BETWEEN-LINEAGE DIFFERENCES IN AVIAN BASE COMPOSITION - WEBER ET AL. (2014)

Supplementary file 6

Estimates of reconstructed ancestral age of first female sexual maturity for a given branch are highly correlated between Coevol chains run on different concatenated sequence alignments. Plots show pairwise correlations, with the x and y axes representing age of 1st maturity for the labelled alignment and rho denoting Spearman's rank correlation coefficient. These results indicate that the alignments were sufficiently long to give reproducible results.

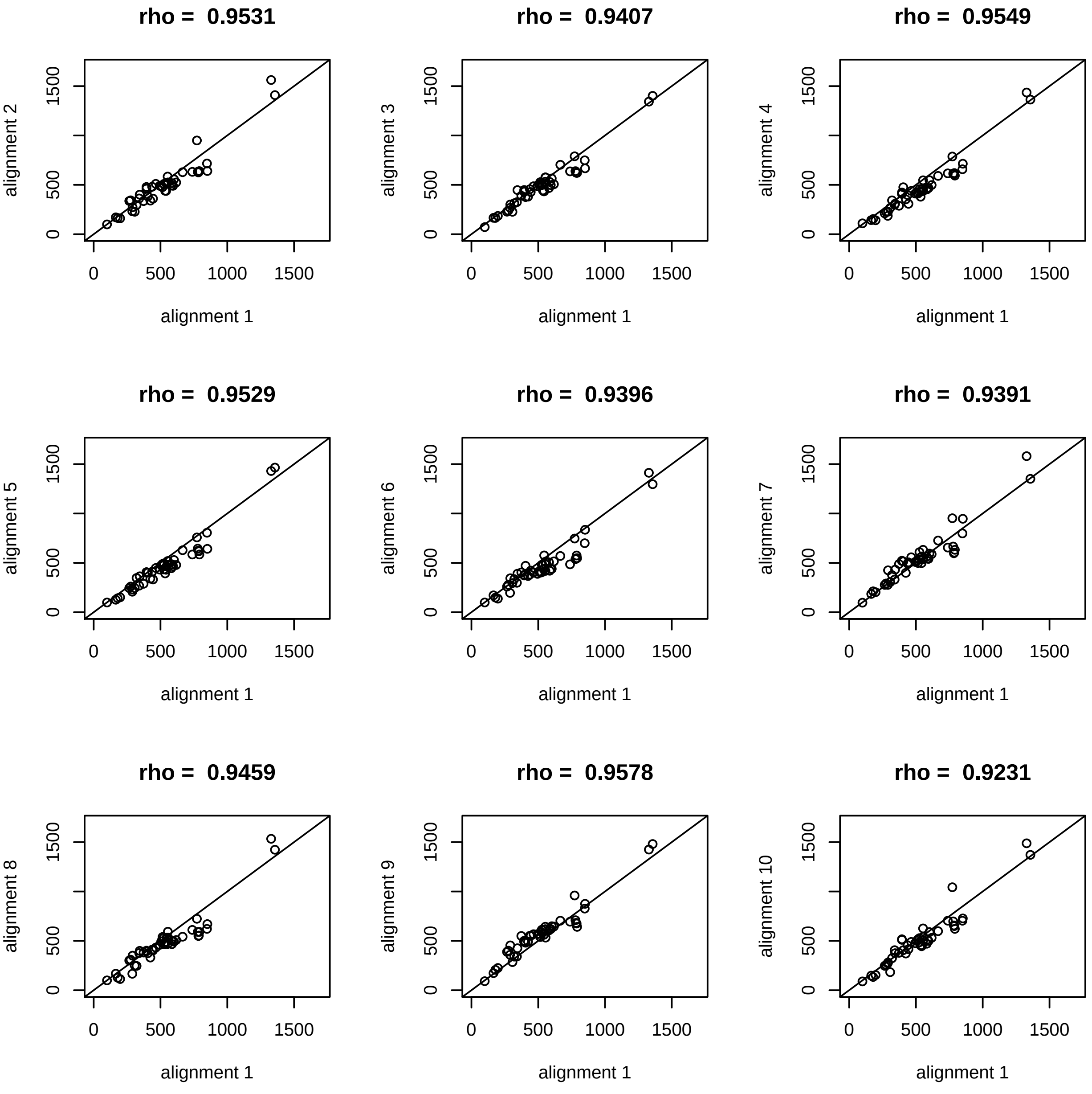
rho = 0.9531
alignment 2
alignment 1
rho = 0.9407
alignment 3
alignment 1
rho = 0.9549
alignment 4
alignment 1
rho = 0.9529
alignment 5
alignment 1
rho = 0.9396
alignment 6
alignment 1
rho = 0.9391
alignment 7
alignment 1
rho = 0.9459
alignment 8
alignment 1
rho = 0.9578
alignment 9
alignment 1
rho = 0.9231
alignment 10
alignment 1
0
500
1000
1500

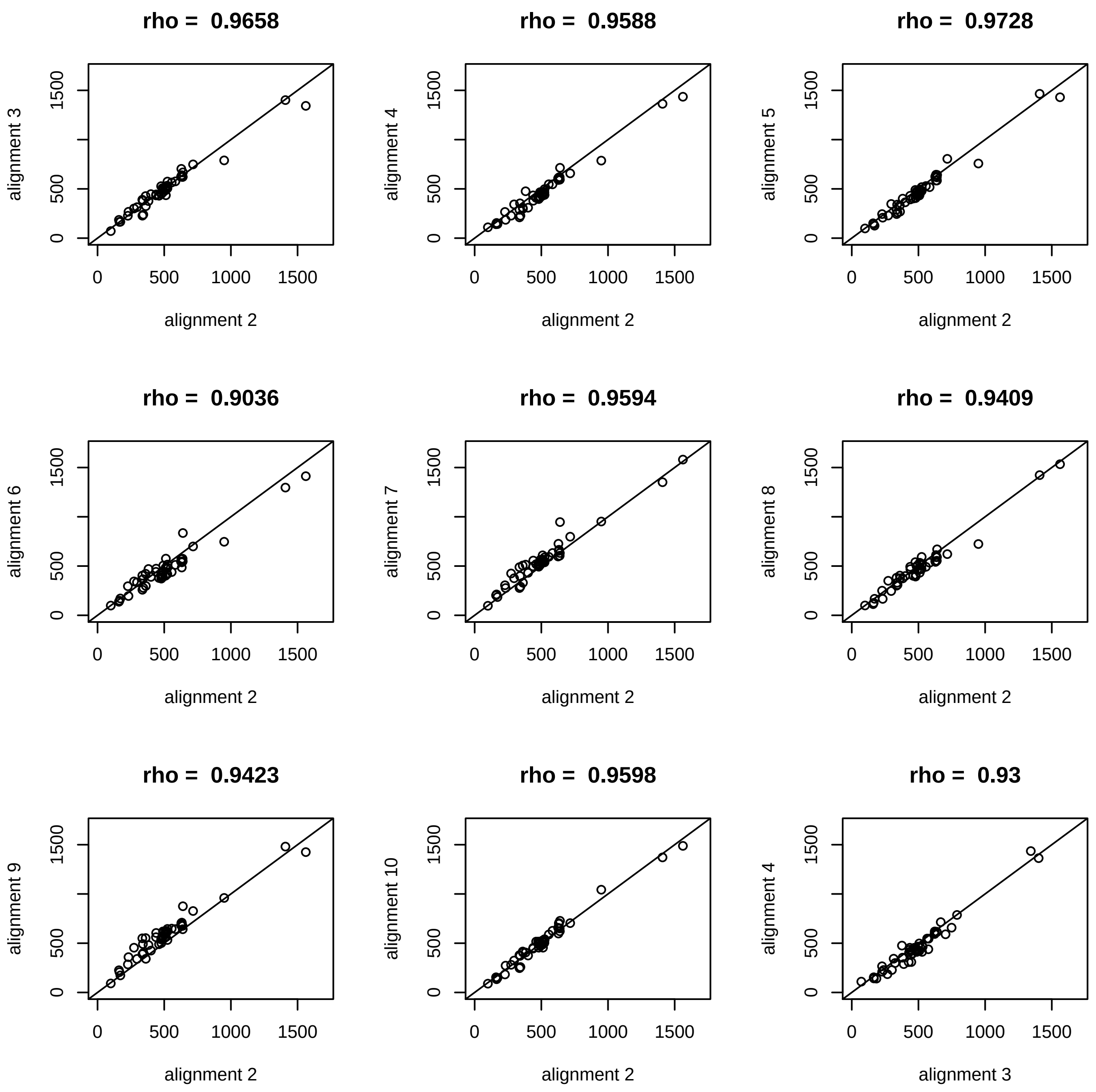
rho = 0.9658
alignment 3
alignment 2
rho = 0.9588
alignment 4
alignment 2
rho = 0.9728
alignment 5
alignment 2
rho = 0.9036
alignment 6
alignment 2
rho = 0.9594
alignment 7
alignment 2
rho = 0.9409
alignment 8
alignment 2
rho = 0.9423
alignment 9
alignment 2
rho = 0.9598
alignment 10
alignment 2
rho = 0.93
alignment 4
alignment 3
0
500
1000
1500

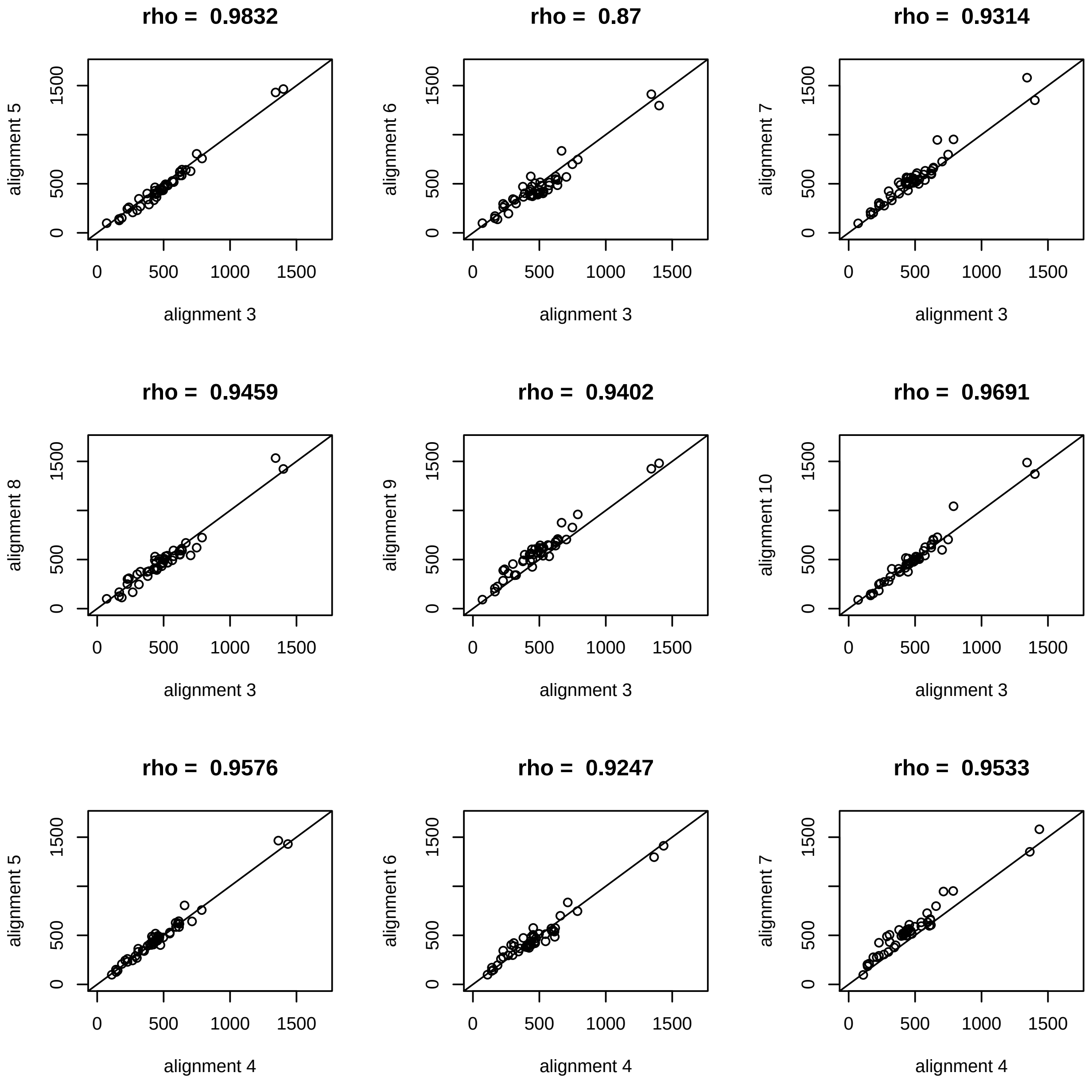
rho = 0.9832
alignment 5
alignment 3
rho = 0.87
alignment 6
alignment 3
rho = 0.9314
alignment 7
alignment 3
rho = 0.9459
alignment 8
alignment 3
rho = 0.9402
alignment 9
alignment 3
rho = 0.9691
alignment 10
alignment 3
rho = 0.9576
alignment 5
alignment 4
rho = 0.9247
alignment 6
alignment 4
rho = 0.9533
alignment 7
alignment 4
0
500
1000
1500

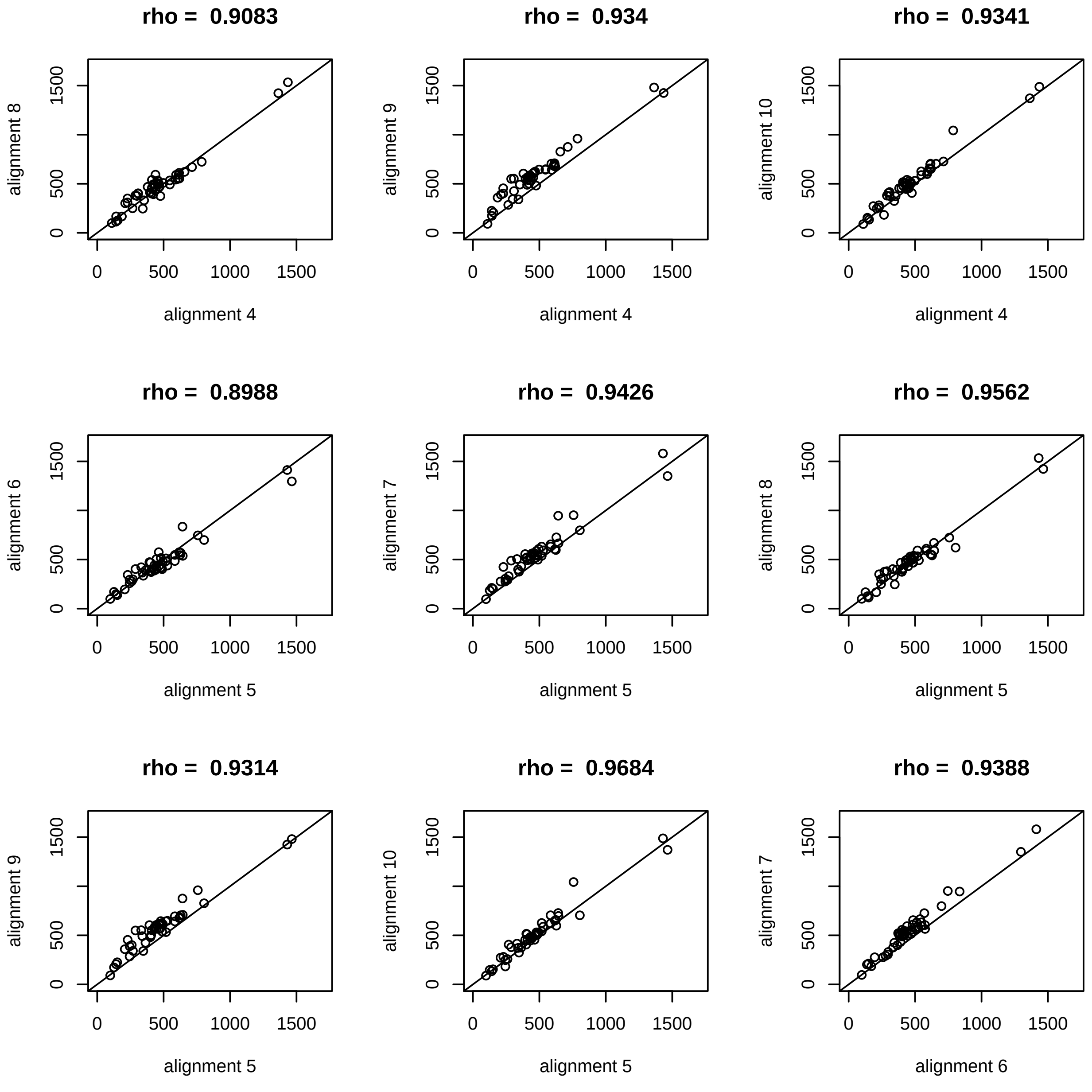
rho = 0.9083
alignment 8
alignment 4
rho = 0.934
alignment 9
alignment 4
rho = 0.9341
alignment 10
alignment 4
rho = 0.8988
alignment 6
alignment 5
rho = 0.9426
alignment 7
alignment 5
rho = 0.9562
alignment 8
alignment 5
rho = 0.9314
alignment 9
alignment 5
rho = 0.9684
alignment 10
alignment 5
rho = 0.9388
alignment 7
alignment 6
0
500
1000
1500

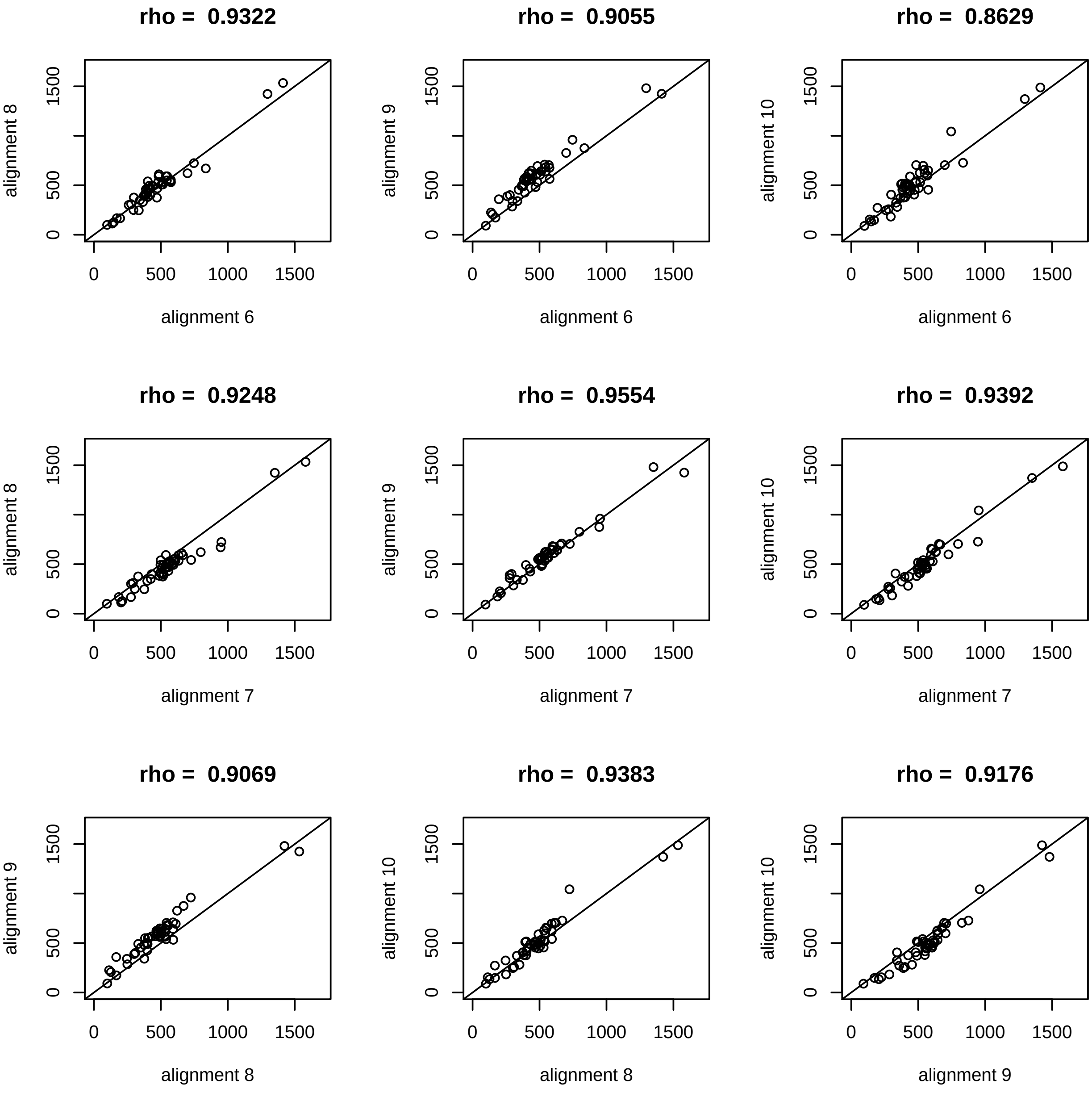
rho = 0.9322
alignment 8
alignment 6
rho = 0.9055
alignment 9
alignment 6
rho = 0.8629
alignment 10
alignment 6
rho = 0.9248
alignment 8
alignment 7
rho = 0.9554
alignment 9
alignment 7
rho = 0.9392
alignment 10
alignment 7
rho = 0.9069
alignment 9
alignment 8
rho = 0.9383
alignment 10
alignment 8
rho = 0.9176
alignment 10
alignment 9
0
500
1000
1500
