# Supplementary material for: Evidence for GC-biased gene conversion as a driver of between-lineage differences in avian base composition
Source: Genome Biol. 2014 Dec 11;15(12):549. doi: 10.1186/s13059-014-0549-1 (PMC4290106; doi:10.1186/s13059-014-0549-1)
Supplement: Additional file 7: — Species pairs used for GC3 conservation analysis. [file 13059_2014_549_MOESM7_ESM.pdf]

EVIDENCE FOR GC-BIASED GENE CONVERSION AS A DRIVER OF BETWEEN-LINEAGE DIFFERENCES IN AVIAN BASE COMPOSITION - WEBER ET AL. (2014)

Supplementary file 7

Species 1	Species 2
Meleagris gallopavo	*Gallus gallus*
Merops nubicus	*Picoides pubescens*
Apaloderma vittatum	*Buceros rhinoceros*
Leptosomus discolor	*Colius striatus*
Cathartes aura	*Haliaeetus albicilla*
Taeniopygia guttata	*Geospiza fortis*
Corvus brachyrhynchos	*Manacus vitellinus*
Melopsittacus undulatus	*Nestor notabilis*
Falco peregrinus	*Cariama cristata*
Egretta garzetta	*Pelecanus crispus*
Nipponia nippon	*Phalacrocorax carbo*
Aptenodytes forsteri	*Pygoscelis adeliae*
Gavia stellata	*Fulmarus glacialis*
Eurypyga helias	*Phaethon lepturus*
Balearica regulorum	*Charadrius vociferus*
Calypte anna	*Chaetura pelagica*
Chlamydotis macqueenii	*Tauraco erythrolophus*
Mesitornis unicolor	*Pterocles gutturalis*
Phoenicopterus ruber	*Podiceps cristatus*
